# The plasticity of pancreatic cancer stem cells: implications in therapeutic resistance

**DOI:** 10.1007/s10555-021-09979-x

**Published:** 2021-08-28

**Authors:** Kalyani Patil, Farheen B. Khan, Sabah Akhtar, Aamir Ahmad, Shahab Uddin

**Affiliations:** 1 Translational Research Institute, Academic Health System, Hamad Medical Corporation, P.O. Box 3050, Doha, Qatar; 2 Department of Biology, College of Science, The United Arab Emirates University, PO Box 15551, Al Ain, United Arab Emirates; 3 Dermatology Institute, Academic Health System, Hamad Medical Corporation, Doha, Qatar; 4 Laboratory Animal Research Center, Qatar University, Doha, Qatar

**Keywords:** Pancreatic cancer, Drug resistance, Cancer stem cells, Epithelial to mesenchymal transition, Oncogenic signaling

## Abstract

The ever-growing perception of cancer stem cells (CSCs) as a plastic state rather than a hardwired defined entity has evolved our understanding of the functional and biological plasticity of these elusive components in malignancies. Pancreatic cancer (PC), based on its biological features and clinical evolution, is a prototypical example of a CSC-driven disease. Since the discovery of pancreatic CSCs (PCSCs) in 2007, evidence has unraveled their control over many facets of the natural history of PC, including primary tumor growth, metastatic progression, disease recurrence, and acquired drug resistance. Consequently, the current near-ubiquitous treatment regimens for PC using aggressive cytotoxic agents, aimed at ‘‘tumor debulking’’ rather than eradication of CSCs, have proven ineffective in providing clinically convincing improvements in patients with this dreadful disease. Herein, we review the key hallmarks as well as the intrinsic and extrinsic resistance mechanisms of CSCs that mediate treatment failure in PC and enlist the potential CSC-targeting ‘natural agents’ that are gaining popularity in recent years. A better understanding of the molecular and functional landscape of PCSC-intrinsic evasion of chemotherapeutic drugs offers a facile opportunity for treating PC, an intractable cancer with a grim prognosis and in dire need of effective therapeutic advances.

## Introduction

Pancreatic cancer (PC) is one of the most aggressive recalcitrant malignancies and portends a high mortality rate [1]. Based on the National Cancer Institute 2021 estimates, PC accounts for 3.2% of all new cancer cases and 7.9% of all cancer-related deaths [2]. In contrast to the steady increase in relative survival for most cancers, advances have been slow for PCs that present a 5-year relative survival of 10.8% [2]. These alarming statistics are ascribed to untimely diagnosis and metastatic organotropism (to the liver and lungs) that often results in the failure of surgical resection, the only clinical method with a potential benefit to PC patients. Despite tangible advances in our understanding of the etiology of PC in recent years, precision medicine has met with little clinical success, largely due to the lack of reliable prognostic/predictive biomarkers that can help to accurately stratify tumors and guide clinical decision-making in patients [3]. Furthermore, efforts have been made to phenotypically stratify pancreatic tumors at the transcriptional level [3]; yet, tumor multifocality, clinical variability, and transcriptomic diversity have stalled the progress in achieving diagnostic, prognostic, and therapeutic breakthroughs. In comparison to other solid malignancies, the mainstay in the treatment of PC is still the conventional chemotherapy, involving the gemcitabine (GEM) plus nab-paclitaxel (NabP) combination (GEM/NabP) regimen. However, de novo and/or acquired resistance to chemotherapeutic drugs is a characteristic feature of PC cells and one of the key reasons that have confounded the efficacy of this systematic treatment. Several potential mechanisms that define the landscape of PC therapeutic resistance, mostly to GEM, have been outlined, including genetic mutations (albeit, poor association), altered metabolism, epigenetic reprogramming, epithelial-to-mesenchymal transition (EMT), aberrant signaling pathways, and the role of tumor microenvironmental components [4]. Still, a detailed understanding of these mechanisms remains largely insufficient. Expectedly, the paramount clinical dilemma in PC management is the development of effective anti-cancer therapeutic strategies that can deal with the complex dynamics of this disease arising from the nonlinear coupling of the evolving genetic diversity, cellular heterogeneity (morphological variations), clonal competition, immune response, metabolic reprogramming, and the tumor microenvironmental interactions.

With the discovery of cancer as a complex adaptive system [5] driven by non-linear dynamics, several theoretical and empirical studies have provided compelling evidence on the coupling between various inducers of tumorigenesis and cancer resistance to therapy [6]. Moreover, multiple genetic and nongenetic mechanisms have been defined that regulate the phenotypic switching of cancer cells to acquire a drug-resistant state within a given cancer type [7]. Over the last decades, cancer stem cells (CSCs; also called “tumor-initiating cells” or TICs) have garnered mammoth attention as critical drivers of drug resistance and tumor recurrence owing to their self-renewing abilities and multilineage differentiation potentials. CSCs possess innately higher chemo- and radioresistance as well as enhanced invasive and metastatic capacity in comparison to their differentiated cancer cell counterparts [8, 9].

Accumulating evidence has confirmed the functional role of highly plastic CSCs in mediating growth, propagation, and chemotherapeutic resistance in PC patients. Pancreatic CSCs (PCSCs), however, exhibit phenotypic and functional diversification that is comparable with interpatient variability detected in primary pancreatic tissue [10]. Owing to this diverse nature, it is plausible that different CSC signatures are associated with relapses and disease progression in PC [11]. Therefore, a systematic examination of CSC heterogeneity, their biological, and functional characteristics is essential to gain better insights into the non-genetic mechanisms that prime drug resistance and tumor relapse in PC.

## Cancer stem cells in pancreatic cancer: association of cell surface markers with tumorigenesis, chemoresistance, and prognosis

Significant advances in delineating pancreatic tumor biology have provided valuable insights into the genetic and epigenetic landscapes associated with the complexity and heterogeneity of PC [12]. Nevertheless, more complex mechanisms underscore the pathobiology of this disease, beyond the genetic-centric paradigm. It is now well-established that tumor heterogeneity emanates intrinsically from diverse subclones endowed with distinct molecular signatures, phenotypic characteristics, and functional roles, such as highly plastic sustainable CSCs [13, 14]. With the introduction of the CSC paradigm, CSCs are *de facto* at the apex of tumor cell hierarchy (constituting < 5% of cancer) and serve as master regulators of tumor progression [15]. Solid pancreatic tumors are hierarchically organized and bear a distinct subpopulation of CSCs [16]. Since the discovery of PCSCs in 2007 [17], numerous studies have confirmed their unique metabolic, autophagic, invasive, and chemoresistance properties. The precise cellular origin of PCSCs is uncertain; however, considering their functional resemblance with the normal stem cell counterparts, it is plausible that PCSCs originate from transformed tissue-specific stem or progenitor cells, bone marrow-derived stem cells (BMDCs), or dedifferentiated cells present in adult tissues formed from genetic mutation [18, 19].

CSC-specific cell surface markers have been extensively used as a tool for their isolation and characterization from various organs. Similar to CSCs, PCSCs exhibit a multitude of markers, such as cluster of differentiation (CD) 44 [17, 20], CD24 [17, 21], CD133 (also known as prominin-1) [22, 23], C-X-C chemokine receptor type 4 (CXCR4) [23, 24], c-Met [25], and epithelial-cell-adhesion molecule (EpCAM; also known as epithelial specific antigen (ESA)) [17]. Still, a detailed understanding of these markers is inadequate or contradictory. Moreover, there is no unison on a “global” signature of molecular markers that can conclusively classify CSCs populations in PC.

Putative PCSCs were first defined as CD44 + CD24 + ESA + subpopulation of PC cells (0.2–0.8%) with enhanced tumorigenic potential [17]. The CD44 + CD24 + ESA + phenotype exhibited a 100-fold increase in tumor-initiating capacity versus non-tumorigenic cancer cells, gauged from a very little number of sorted cells required to produce tumors histologically similar to primary PC in immunocompromised mice. Importantly, the CD44 + CD24 + ESA + cells displayed distinctive stem cell features, including self-renewal, ability to generate phenotypically diverse (differentiated) progeny, and elevated expression of the developmental signaling molecule sonic hedgehog (Shh) [17]. Irrespective of some potential limitations observed in the study, these novel results opened an avenue of research in PCSC biology.

### CD24 

CD24 is a small mucin-like glycosylphosphatidylinositol-anchored membrane protein that functions primarily as an adhesion molecule for P-selectin [26] and L1 [27] and plays a role in B-cell development and neurogenesis. It is also implicated in governing multiple cell properties to favor tumor growth and metastasis. In cellular and animal assays, CD24 works as a pleiotropic stimulator of tumor cell proliferation, adhesion to extracellular matrix (ECM) components, motility, and invasion [28].

Whilst CD24 plays a pivotal role in influencing tumorigenesis, it exhibits diverse functions that primarily depend on tumor entities and its localization to the subcellular compartments [29]. However, there is considerable ambiguity and conflicting data on CD24 classification, distribution, and subcellular localization responsible for eliciting different effects during invasion and metastasis [29]. Research has shown enhanced CD24 expression in pancreatic ductal adenocarcinoma (PDAC; accounting for > 90% of all pancreatic neoplasms) and its association with metastasis [29, 30], but its influence on invasiveness is inconsistent. This inconsistency has been attributed, in part, to the heterogeneous expression and different forms (intracellular and/or cell surface) of CD24 in PDAC. The molecular differences between intracellular and cell surface CD24 are currently unknown, further adding to the discrepancy in the literature [31]. Nevertheless, surface CD24, along with CD44 and ESA expression, has been used for the classification of putative CSCs in PCs [17] and is associated with a significant number of pancreatic intraepithelial neoplasia (PanIN) lesions [32]. Functional analysis in genetically engineered mouse models (GEMM) for PC has identified CD24 as a positive regulator of the Wnt/β-catenin pathway activated during tumor differentiation, with a specific function of surface CD24 in regulating EMT phenotypes [29]. CD24 shows transient surface localization during PDAC development, and this accounts for a few tumor cells that can be isolated expressing surface CD24 [29]. Further investigations into the clinical relevance of CD24 expression in PC have signified its association with higher tumor stages [21], high grade tumors [21], shorter overall survival (OS) [33], and advanced pT stages [21].

### CD133

CD133 is a glycosylated pentaspan protein and a recognized CSC marker in several cancer entities, including PC [22, 23, 34]. CD133 regulates an array of cell signaling pathways, including Akt, B-cell lymphoma 2 (Bcl-2), Src, Ras, and its downstream effectors such as extracellular signal-regulated kinase (ERK), c-Jun N-terminal Kinase (JNK), phosphoinositide 3-kinases (PI3K), signal transducer and activator of transcription (STAT) 3, and p38K [35, 36]. It is also engaged with the Notch pathway, connected to dysregulated cell cycling and drug resistance [37, 38] as well as with Shh facilitating anchorage-independent growth [39]. Additionally, CD133 physically associates with histone deacetylase HDAC6 and β-catenin leading to the formation of a functional module, thus activating Wnt signaling and promoting EMT, cancer cell migration, and metastasis [40]. These interactions are relevant to the critical role of CD133 in the enhancement of stemness, tumorigenicity, and chemotherapeutic resistance [18].

The invasive border zone of pancreatic tumors is enriched with CD133 + CXCR4 + CSC subpopulation capable of reconstituting primary tumor growth with full tumor differentiation in permissive recipients [23]. CD133 + cells display hyperproliferation under anchorage-independent conditions and enhanced migration and invasion, particularly when co-cultured with primary pancreatic stromal cells expressing CXCR4 [41]. A direct correlation between hypoxia and CD133 expression has been established; CD133 + cells co-localize to the hypoxic areas within the pancreatic tumors and show enhanced hypoxia-inducible factor-1α (HIF-1α) activity. Under hypoxia, PC cells acquire stem-like phenotypes through the expansion of CD133 + subpopulation, consequently leading to an aggressive phenotype and increased invasiveness predominantly in a HIF-1α -dependent manner [42]. Overexpression of CD133 in cultured human PC cell line MIA PaCa-2, bearing only 0.1% endogenous CD133, induces stemness properties via upregulating stemness genes KIT ligand (KITLG), Lin-28 Homolog B (LIN28B), c-Myc, Kruppel-like factor 4 (KLF4), Gli1, Sox2, Nanog, sirtuin 1 (SIRT1), POU Class 5 Homeobox 1 (POU5F1), and CXCR4. Functionally, CD133 overexpression increases dye efflux and aldehyde dehydrogenase (ALDH) activity which are the characteristic features of authentic CSCs [43]. Furthermore, overexpressed CD133 increases tumorigenic potential by the induction of nuclear factor kappa-light-chain-enhancer of activated B cells (NF-κB) pathway activation [43]. Along with direct stimulation of HIF-1α expression, the CD133-NF-κB-HIF axis is considered another mechanism that regulates HIF-1α mRNA expression in hypoxic conditions [44]. CD133-induced CSC activity is also attributed to the enhanced expression of telomerase reverse transcriptase favoring cellular immortalization and CD133 ligand-independent epidermal growth factor receptor (EGFR) activation [45].

The influence of CD133 + PCSCs on the drug-resistant phenotype is mainly attributed to its metabolic plasticity adopted in response to the stress induced from the increased generation of reactive oxygen species (ROS) [46]. This altered metabolic profile seems to offer a survival advantage to CD133 + PCSCs in conditions of increased ROS accumulation that is induced by cytotoxic concentrations of 5-fluorouracil (5-FU), GEM, and Paclitaxel [47]. In addition to altered bioenergetics, increased ATP-​Binding Cassette (ABC) transporter activity coupled with elevated expression of classic apoptosis regulators Bcl-2 and Survivin in CD133 + PCSCs [48] also contributes to their chemoresistant phenotype. Given the role of HIF1 in controlling the balanced expression of ABC transporters [49], it is speculated that elevated levels of HIF1A in CD133 + subset modulates the expression of transporter proteins leading to the increased efflux of chemotherapeutic agents.

With respect to the clinicopathological features, previous studies have related CD133 overexpression with the clinical TNM stage, poor differentiation, lymph node metastasis, and a lower survival rate in PC patients [50, 51]. Through multivariate analysis, high co-expression of CD44/CD133 in PCSCs was identified as an independent prognostic factor for disease-free survival [52]. However, recent data has highlighted its clinical insignificance in PDAC as a CSC marker indicative of tumor stage or disease activity. It is hypothesized that CD133 expression could represent the cells of possible CSC potential that might be prone to malignant transformation [53]. Therefore, more detailed studies on the clinical relevance of CD133 is required.

### CXCR4

The chemokine network, involving a superfamily of intercellular signaling proteins, regulates an array of biological processes, such as embryogenesis, organogenesis, and tissue homeostasis [54]. Although majorly involved in immune responses, chemokine/chemokine receptor systems have also been assigned several extra-immunological functions [55], particularly in malignancies, where it influences the tumor cell growth, survival and migration, angiogenesis, and metastasis [56].

Of the various chemokine signaling networks, the C-X-C Motif Chemokine Ligand 12 (CXCL12)/CXCR4 axis is recognized as a prominent moderator of the supportive tumor microenvironment (TME) and tumor-stroma interactions [57, 58]. CXCR4, a G-protein coupled receptor (GPCR) [59], is one of the most ubiquitously overexpressed chemokine receptors in diverse cancers and in conjunction with its primary chemokine ligand, CXCL12 (also known as stromal-derived factor-1, SDF-1), impacts several hallmarks of cancer including resistance to apoptosis, sustaining proliferative signals, angiogenesis, evading growth suppression, replicative immortality, and invasion and metastasis [58].

In PDAC, this chemokine axis is directly implicated in invasion and metastasis, partly via its crosstalk with key oncogenic signaling pathways such as Akt, ERK, c-myc, β-catenin, NF-κB, and p53 [58]. Specific to PCSCs, a subpopulation of migrating CD133 + CXCR4 + CSCs has been detected that is associated with the invasive and metastatic profile of PDAC [23, 60]. In vivo experiments using sorted CD133 + CXCR4 + cells have affirmed the significance of CXCR4 co-expression in markedly increasing the migratory activity of metastasizing CSCs and generating liver metastasis [23]. Re-expression of CXCR4 following dedifferentiation of the ductal epithelium into stem cell-like phenotype during carcinogenesis promotes cancer cell survival [58]. CXCR4 activation contributes to the chemoresistant signature of pancreatic tumors by augmenting the production of Shh which, in an autocrine fashion, promotes EMT and a more stem cell-like state of PC cells [61]. Secreted Shh, in turn, modifies the fate and behavior of pancreatic stellate cells (PSCs; a specialized type of cancer-associated fibroblast (CAF)) in the stroma that further participate in the positive feedback system to boost tumor growth [58]. Recent analysis has also shown an indispensable role of the CXCR4/let-7a/HMGA2 pathway in tumor-associated phenotypes and chemoresistance of PC cells to GEM [62]. From a clinical perspective, enhanced CXCR4 tumor expression is associated with poor prognosis, lower 5-year OS, and a greater chance of developing lymph node metastasis and liver recurrence in patients afflicted with PC [24, 58].

### c-Met

c-Met is a MET proto-oncogene receptor tyrosine kinase (RTK), abnormal stimulation of which actuates an ‘invasive growth’ program in cancer cells [25]. Upon interactions with its specific ligand hepatocyte growth factor (HGF), the c-Met signal is relayed downstream to stimulate a series of signaling pathways in tumor cells, such as PI3K/Akt, Janus kinase (JAK)/STAT, Ras/mitogen-​activated protein kinase (MAPK), Src, and Wnt/β-catenin [63, 64], exerting control over tumor proliferation, apoptosis resistance, EMT, angiogenesis, invasion, and metastasis [65–68].

Aberrant HGF/c-Met axis activation, which is closely related to c-Met gene mutations, overexpression, and amplification, occurs in a variety of solid organ neoplasms including PC [69]. In pancreatic neoplasms, the HGF/c-Met axis is involved in the intricate tumor-stroma crosstalk [70], GEM-resistance *in vivo* [71], and metastasis of therapy-resistant tumor cells [72]. Evidence has also highlighted the essential role of HGF/c-Met signaling in the maintenance of pancreatic progenitors and stem cells [73].

c-Met has long been recognized as a putative PCSC marker with crucial functions in PCSC biology. The association between c-Met and stemness of PC cells was first established by Li et al. using a NOD/SCID mouse xenograft model [25]. The team identified c-Met^HI^ PCSC population exhibiting increased tumorigenic potential and self-renewal capacity than c-Met^−^ cells. They reported that cells with a c-Met^HI^CD44^+^ marker profile represent a highly tumorigenic population with characteristic stem cell behavior, including self-renewal and the ability to phenotypically recapitulate parental tumor. Using c-Met inhibitor XL184 or knockdown by small hairpin RNAs, a functional role of c-Met in maintaining PCSC survival and function was also determined. Another group has demonstrated the susceptibility of c-Met^HI^ population to epigenetic reprogramming by core reprogramming factors c-Myc, Oct4, Sox2, and KLF4 [71]. This suggests that c-Met plays a functional role in maintaining CSC properties including reprogramming and epigenetic modification of malignant features of PCSCs.

In the clinical scenario, c-Met overexpression represents an adverse prognostic marker in patients with PDAC, with a direct correlation to tumor grade, increased tumor-node-metastasis stage [69], and poor survival [74]. Cumulatively, the functional role of c-Met in PCSCs and tumor behavior in PDAC has made it an attractive target of consideration while designing effective treatment regimens against PC.

### CD44/CD44v6

CD44, a non-kinase transmembrane adhesion receptor that binds ECM hyaluronan (HA), is a bonafide molecular marker of CSCs [75]. This ubiquitous transmembrane molecule is preferentially upregulated in a range of tumors, particularly, in TICs and drug-resistant tumor lesions [76]. During tumorigenesis, CD44 undergoes extensive alternative splicing generating two isoforms with overlapping and distinct cellular functions: the CD44 variant (CD44v) and CD44 standard (CD44s) isoform [77]. Although the functional significance of distinct CD44 isoforms in the pathogenesis of cancer is under investigation, the dysregulation of isoform switching has been determined [77] and implicated in regulating EMT and the adaptive plasticity of cancer cells [75], potentially generating adaptive therapeutic resistance and tumor recurrence [78].

The phenomenon of CD44 splice isoform switching in PC has been illustrated by Zhao et al. [79]. The authors identified a highly invasive, metastatic, mesenchymal-like subpopulation of PDAC cells expressing high levels of CD44s isoform (CD44s/EMT) and stem cell-like properties which eventually induce the formation of GEM-resistant tumors exhibiting a CD44 isoform switch into the variant isoform. Notably, CD44^HI^ PC tumors, initially responsive to GEM, gradually developed resistance after 12 weeks of treatment, whereas CD44^LOW^ tumors showed apparent sensitivity through 22 weeks of therapy [79]. This observation suggests that CD44 may serve as a predictive biomarker for chemoresistance, providing knowledge on the time taken to develop resistance.

Several investigations into the mechanistic relationship between drug resistance and CSCs have highlighted a crucial role of the ABC superfamily of transporter proteins in the detoxification of xenobiotics and anti-tumor drugs in PC [80]. Overexpression of three proteins belonging to the ABC transporter superfamily has been identified in CSCs and extensively studied in PC, including P-glycoprotein (P-gp, also known as ABCB1 or multidrug resistance (MDR) protein 1 (MDR1)), breast cancer resistance protein (BRCP or ABCG2), and the MDR-associated protein 1 (MRP1 or ABCC1) [80]. These three transporter proteins possess a broad substrate specificity and overlapping drug specificity and have been associated with worse responses to an array of chemotherapeutic drugs [80, 81]. Among these transporter proteins, the significant overexpression of ABCB1 was found to be concomitant with the proliferation of resistant CD44 cells, suggestive of the regulatory role of CD44-ABCB1 interaction in GEM efflux in pancreatic tumor cells [20]. Recent investigation has also found overexpression of pancreatic adenocarcinoma up-regulated factor (PAUF) in CD44 + CD24 + ESA + PCSCs that attributes to both GEM and 5-FU resistance by increasing the mRNA expression of ATP-dependent multidrug-resistant protein 5 (MRP5, ABCC5) and ribonucleotide reductase regulatory subunit M2 (RRM2) [82]. Besides, PAUF has been shown to exert control over the expression of stemness genes (Oct4, Nanog, and Sox2), and other CSC markers (such as CD133, and c-Met) [82].

Characterization of the molecular mechanisms underlying acquired resistance to GEM downstream from the drug-target interaction has also identified overexpression of CD44, together with the upregulation of c-Met and STAT3 and downregulation of total and phosphorylated Src. In addition, hyperactive EGFR following increased autocrine production of its ligand amphiregulin (AREG) has been detected in PC drug-resistant variants [83]. Intact autocrine EGFR signaling cascade, induced by the redox master regulator Nuclear factor erythroid-derived 2-like 2 (Nrf2/Nfe2l2) through Akt [84], is an important adaptive survival response that contributes to drug resistance in Kras mutant cancer cells [85]. Redox regulation by Nrf2 has been shown to support PDAC initiation and maintenance by modulating mRNA translation and mitogenic signaling in cancer cells [84]. In response to GEM-induced generation of ROS in PC cells, activation of Nrf2 causes an increase in glutathione (GSH) and heme oxygenase 1 (HO-1) levels that lowers intracellular ROS concentration and prevents ROS-induced DNA damage [84, 86]. HO-1 knockdown or inhibition by zinc protoporphyrin and tin protoporphyrin IX (SnPP) has been demonstrated to suppress the proliferation of PDAC cells under hypoxia, reduce expression of CD44, and sensitize them to GEM in vitro [87]. It is plausible that the prevention of ROS-related damage to PC cells following GEM therapy is related to the stemness properties and specifically to CD44 + CSCs in PC.

Besides the predominance of standard isoform, EMT-ed PDAC cells also express small molecular size exon CD44 variants CD44v3 or CD44v6 [79]. CD44v6 is the most widely studied CD44 variant form in PC and is frequently upregulated in cells with high metastatic potential and stem cell-like characteristics [88, 89]. Of central importance in understanding the contribution of CD44/CD44v6 in CSC activities is its crosstalk with RTK complexes, GPCRs, integrins, cytosolic signaling molecules, proteases, and cytoskeletal linker proteins [90]. One such example that highlights the co-receptor function of CD44, for RTK complexes, is the identification of a highly tumorigenic, stem-like population of PC cells marked with c-Met^HI^CD44^+^ expression, as discussed earlier [25]. The significance of the CD44 co-receptor function has also been implied in the growth and maintenance of metastasis in pancreatic tumors. Matzke-Ogi et al. demonstrated increased CD44v6 mRNA levels in human pancreatic tumor tissues and its association with increased expression of c-Met and tumor metastasis [91]. In fact, CD44v6 is implicated in organizing an integral signaling hub for PC metastasis [91]. CD44v6/v9 double-positive pancreatic tumors are linked to metastasis and lower OS [92]. Clinical analysis has shown the correlation of CD44v6 + expression with lymph node metastasis, liver metastasis, TNM stage, and shorter patient survival times [92]. Recently, high CD44 H-scores, together with high glycan carbohydrate/cancer antigen 19–9 (Ca19-9) levels and poor differentiation, were proposed to be independent predictors for early recurrence in PDAC patients undergoing radical resection [93].

### EpCAM

EpCAM is a type I epithelial transmembrane glycoprotein and a homophilic Ca2 + -independent cell–cell adhesion molecule [94]. EpCAM exhibits a broad functional spectrum in multiple physiological, developmental, and pathological processes [95]. It contributes to the homeostatic maintenance of epithelial tissues via the regulation of cell–cell junctions, signaling pathways, cellular proliferation, polarity, and mobility [95]. Besides developmental processes, EpCAM is upregulated or de novo expressed in the majority of epithelial tumor tissues and derived metastasis, including PC [96, 97]. This may relate to its active role in regulating proliferation and metabolism of epithelial cells and fibroblasts via a rapid induction of the proto-oncogene c-Myc and the cell cycle regulating genes cyclin A and E [98]. Further evidence supporting EpCAM influence on cell proliferation comes from the positive correlation between EpCAM expression and cell cycle progression via control on cyclin D1 expression and direct interaction with four-and-a-half LIM domains protein 2 (FHL2) [99]. In vivo and in vitro studies have also established (partial) connection of EpCAM expression to EMT in PC [100]. Although counted as one of the CSC markers, there is limited information on whether EpCAM fulfills CSC-specific tasks. EpCAM + PCSCs have been shown to possess enhanced tumorigenic potential compared with EpCAM- PC cells [17]. Additionally, EpCAM has been demonstrated to inhibit tumor-infiltrating immune cells through an interaction with its extracellular ligand, leukocyte-associated immunoglobulin-like receptor (LAIR1) [98], thus explaining the underlying mechanism of active immune escape mechanisms in EpCAM-expressing tumors. Despite this compelling evidence supporting the tumor-promoting role of EpCAM, its anti-tumorigenic effects have also been noted [101].

Similarly, the clinical significance of EpCAM and its influence on clinical prognosis is also a matter of debate. Some clinical reports have associated high EpCAM expression with a good prognosis [102, 103], whereas other studies have identified high EpCAM expression as a factor for poor prognosis [104, 105]. Such discrepancy suggests that EpCAM may have a different role in each type of cancer. It is speculated that EpCAM’s prognostic value depends on the tumor entity. In patients with advanced PC, EpCAM overexpression relates to poor prognosis [106] and a shorter survival period of 48 months as against 70 months without this marker [107]. Contrarily, EpCAM expression relates to good prognosis in PC patients receiving the curative resection, ascribed to its suppressive effects on PC cell activity [100] (Table 1).

**Table. 1:** Pancreatic CSC markers and their functional relevance

Stem cell marker	CSC phenotype	Resistance to Gemcitabine	Signaling pathways involved	Role in pancreatic cancer (cells)	Pancreatic cancer features associated with high expression	Prognosis
CD24	CD24 + CD44 + ESA +	Resistant[17]	Hedgehog signaling pathway [17]	Tumor differentiation [29]	High-grade tumor [21]	Shorter overall survival [21]
			Wnt/β-catenin pathway [29]	Regulation of EMT [29]	Higher tumor stage [21]	
			Notch pathway [108]	Invasiveness and Metastasis [29, 30]	Nodal metastasis [21]	
CD44	CD44 +	Resistant [17, 79]	Crosstalk with RTK complexes [25]	Increased GEM efflux via ABC transporter proteins [20]	Lymph node metastasis [92]	Lower overall survival [92]
	CD44 + CD24 + ESA +		Notch signaling [109]	Induction of EMT [79, 110, 111]	Liver metastasis [92]	
				Increased metastatic potential [91]	Advanced TNM stage [92]	
					High Ca19-9 levels [93]	
					Poor differentiation [93]	
CD133	CD133 + CXCR4 +	Resistant [112]	Hedgehog signaling pathway[39]	Tumor differentiation [23]	Higher tumor stage (?)	Lower survival rate [50, 51]
			Notch pathway[37, 38]	Acquisition of stemness [42, 43]	Lymph node metastasis [51]	
			NF-κB pathway [43]	Invasion and metastasis [43, 113]		
			CD133-NF-κB-HIF signaling axis [44]	Aggressive phenotype and increased invasiveness in hypoxia [42]		
				Increased drug efflux [43, 48]		
				Induction of EMT [43, 114]		
				Altered bioenergetics [48]		
				Apoptosis resistance [48]		
CXCR4	CXCR4 + CD133 +	Resistant [61]	CXCL12/CXCR4 axis	Metastasis [43]	Lymph node metastasis [24, 58]	Poor prognosis[24, 58]
			Hedgehog pathway [61]	Induction of EMT [61]	Liver recurrence and metastasis [23, 24, 58]	Shorter overall survival[24, 58]
			CXCR4/let-7a/HMGA2 pathway[62]	Enhanced invasive and metastatic profile [60]		
			Crosstalk with Akt, ERK, c-myc, β-catenin, NF-κβ, and p53 [58]	Acquisition of stem-like phenotype [58, 61]		
EpCAM	CD44 + CD24 + ESA +	Resistant [17]	Induction of c-Myc and cyclin A and E [98]	Regulation of cell proliferation and metabolism [98]	Lymphatic spread [115]	Shorter survival [107]
			MAPK and JNK activation [115]	Enhanced tumorigenicity [17]		Prognosis (?)
				Immune escape [98]		
				Regulation of EMT [100]		
				Apoptosis resistance [115]		
DCLK1	DCLK1 +	ND	Notch pathway[116]	Acquisition of hypoxia-induced stemness [117]	Liver metastasis [118]	ND
			Hippo pathway [119]	Evasion of immune surveillance viaPD-L1 [119]		
			ABL1 and IGF1R pathway [116]	Repression of tumor suppressor miRNAs let-7a, miR-144, miR-200a-c, and miR-143/145 [120]		
			Regulation of pluripotency genes [120]	Regulation of EMT and angiogenesis [120–122]		
				Enhanced invasive and metastatic potential [116, 118]		
c-Met	c-MetHighCD44 +	Resistant [123]	HGF/c-Met signaling axis	Maintenance of PCSC survival and function [25]	Invasion and metastasis [69, 72]	ND
				Involvement in tumor-stroma crosstalk [70]		

*ND* not determined; (?) represents inconclusive

## Other potential pancreatic stem cell markers

Salient developments in profiling CSCs based on their physiological and functional properties in PC has expanded the directory of inherent CSC markers such as overexpression of core stem cell transcription factors Oct4, Sox2, and Nanog [124], expression of doublecortin and Ca2 + /calmodulin-dependent kinase-like 1 (DCLK1) [125], and that of cell surface receptor leucine-rich repeat-containing G protein-coupled receptor 5 (Lgr5) [126]. 

DCLK1 has recently gained widespread recognition as a CSC marker in the pancreatic, colon, and other cancers [127], besides being an accepted tuft cell marker in the small intestine [127, 128]. Studies have identified morphologically and functionally distinct subpopulations of tumor-initiating PC cells, in preinvasive (PanIN) and invasive pancreatic neoplasms, marked by the expression of DCLK1 and CSC-like properties [116]. Microarray and siRNA screening assays have shown predominant expression of DCLK1 with H3K4me3 and H3K27me3 histone modification in PCSCs with invasive and metastatic potential [118]. Overexpressed DCLK1 is associated with amoeboid morphology in PCSCs that enhances their migration and the ability to form liver metastasis [118]. DCLK1 expression has also been found to significantly correlate with CD44 + /CD24 + /EpCAM + expression as well as EpCAM expression in PDAC cells [129]. Whole transcriptome analysis of genes and pathways potentially modulating the tumor-initiating capacities and clonogenic functions of DCLK1^HI^/acetylated α-tubulin (AcTub^HI^) PDAC cells have revealed upregulation of tuft cell markers (TAS2R31, OR5A2), tubulin acetylation enzyme (ATAT1), Notch response genes (HES1, HES7, and HEY1), proto-oncogene ABL Proto-Oncogene 1 (ABL1), and insulin-like growth factor 1 receptor (IGF-1R) [116]. According to a recent study, increased expression of DCLK1 assists in hypoxia-induced stemness in pancreatic tumors, initiated by the cooperation between HIF-1α and histone lysine demethylase 3A (KDM3A) [117]. Along with oncogenes, DCLK1 also modulates stem cell pluripotency in PDAC through the regulation of multiple tumor suppressor microRNAs (miRNAs/miRs) such as miR-200, miR-145 (miR143/145 cluster), and let-7a and their downstream pro-tumorigenic pathways [120].

Lgr5, a cell surface-expressed Wnt target gene and a receptor for the Wnt‐agonistic R‐spondins (RSPOs) [130], is a novel bonafide marker of adult organ stem cells [131, 132] as well as a functional biomarker of CSCs [133], contributing to cancer stemness traits through the regulation of Wnt/β-catenin signaling pathway [134, 135]. Several reports have outlined the stimulatory effects of Lgr5 in tumor growth, especially in gastrointestinal cancers, through the regulation of CSC stemness, EMT, and tumor cell proliferation [136]. Regardless, in PC, the expression and functions of Lgr5 are still unclear despite being proposed to be on a higher level of the stem cell hierarchy than CD133 [126, 137]. Only a limited number of studies have examined and evaluated Lgr5 expression in PDAC [138, 139]. Amsterdam et al. identified a stem cell niche in the islets’ β cells of the normal pancreas expressing Lgr5 and Nanog stem cell markers and containing the potential cell-of-origin of PDAC [140]. The team also observed Lgr5 expression in cancerous pancreas in the remaining islets and all ductal cancer cells. Kuraishi et al. found declining Lgr5 expression with tumor progression and dedifferentiation, suggesting that Lgr5 + cells may function as CSCs only in the initial phase of carcinogenesis [141]. Thus, detailed investigations into the exact mechanism by which Lgr5+ cells contribute to the development of PC are required.

Oct4 is a member of the Pit, Oct, Unc (POU) family of DNA binding-proteins and one of the important transcription factors that govern pluripotent embryonic stem cell (ESC) identity across mammalian species [142]. It has been recognized as a master regulator of ESC pluripotency, controlling cell differentiation, somatic cell reprogramming, and renewal [142, 143]. Considering gene expression similarities between CSCs and early ESCs [124] as well as the ability of CSCs to reactivate embryonic programs [144], publications have demonstrated the regulatory role of core stem cell factors Oct4, Sox2, and Nanog, together or separately, in maintaining pluripotency and self-renewal in tumors [145]. In pancreatic tumors, Oct4 and Nanog have been found to influence proliferation, colony formation, migration, invasion, chemosensitivity, and tumor formation capacity of PCSCs by controlling the expression of downstream genes TIMP Metallopeptidase Inhibitor 1 (TIMP1), CXCR4, matrix metalloproteinase (MMP)-2, MMP-9, and ABCG2 [146]. ABCG2 is typically associated with CSC-driven therapy-resistance in clinical applications [147]. Overexpressed ABCG2 has also been identified in side population (SP), derived from human PDAC samples, that are enriched with cells displaying CSC-associated properties and GEM-resistance [148]. Although literature has confirmed the ubiquitous expression of ABCG2 in PDAC cells and its role in bestowing SP phenotype, the extent to which it contributes to the refractory nature of PDAC remains unclear. Bhagwandin et al. identified ABCG2 as a ubiquitous source of drug resistance in PDAC; however, it did not offer resistance to the first-line therapeutic GEM [149]. Nevertheless, tractable inhibitors of ABCG2 have been proposed as useful adjuncts in the treatment regimens targeting CSCs in PDAC.

In the last decade, research has diversified the CSC marker profile and now includes the expression of miRNAs and long noncoding RNAs (lncRNAs) [150, 151], CSC-derived exosomes and their bioactive cargo [152, 153], high 26S proteasome activity [154], and PCSC secretome-associated proteins including fatty acid synthase (FASN), galectin-3, acetoacetyl-CoA transferase (ACAT2), ceruloplasmin, Ca19-9, and myristoylated alanine-rich C kinase substrate (MARCKS) [155, 156]. Regardless, there are conflicting opinions on the use of some of these markers, considering the relatively smaller inter-tumoral or inter-species overlap amongst the CSC markers.

## Dysregulated pancreatic cancer stem cell-related signaling pathways in maintenance and therapy-resistance

**Fig. 1:**
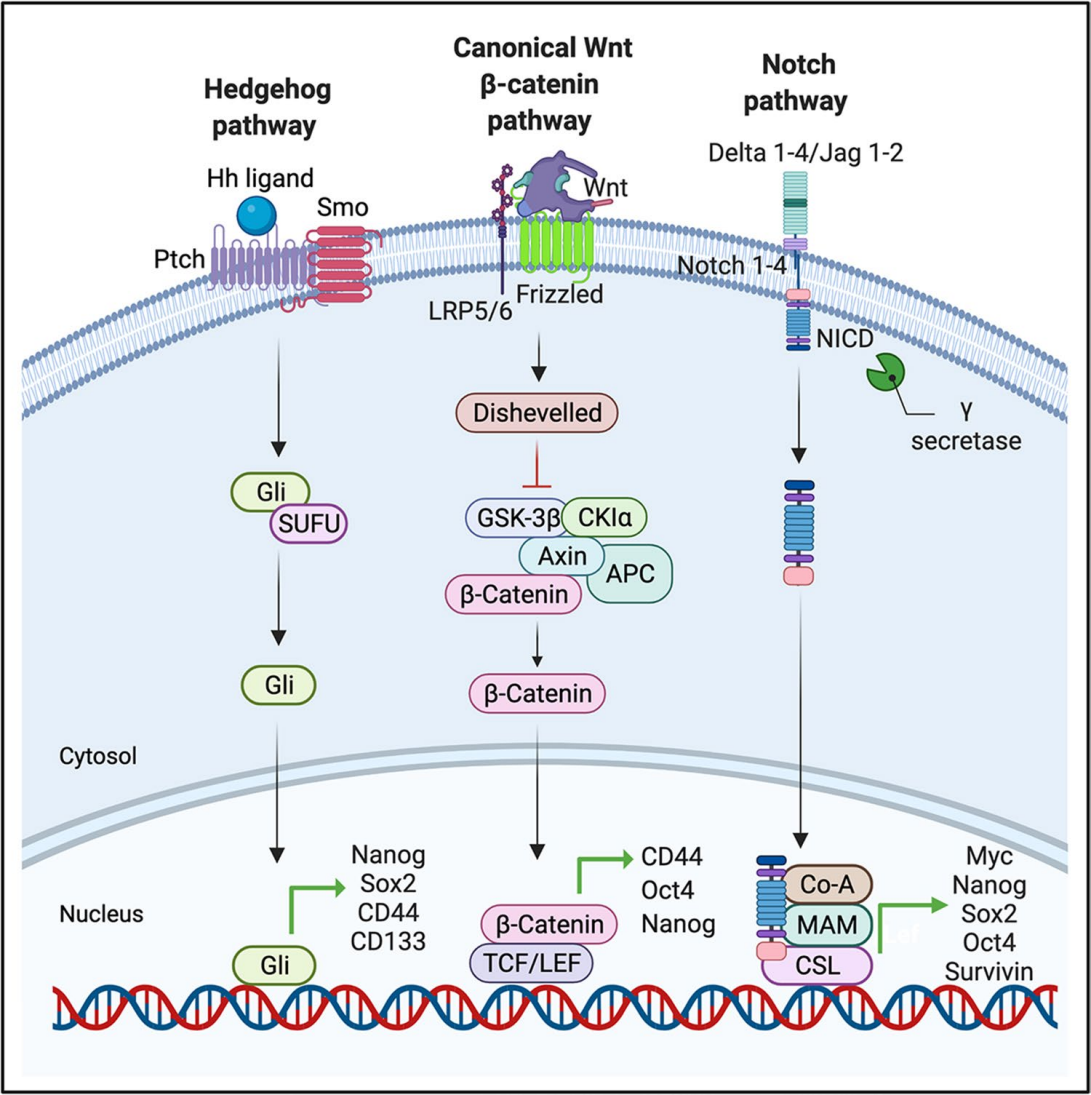
Key signaling pathways regulating CSC state in pancreatic cancer. Amongst an array of signaling pathways aberrantly activated in PCSCs, Notch, Wnt, and Hedgehog pathways are crucial for the maintenance of self-renewal, tumor development, invasion, metastasis, and therapy-resistance. In the canonical Hedgehog pathway, binding of the exogenous Hh ligand to its cognate receptor Ptch removes the inhibitory influence of Ptch on Smo, thereby activating Smo and the downstream Gli proteins, which upon nuclear translocation induces target (stemness) gene expression. The canonical Wnt signaling pathway is activated upon binding of the Wnt ligand to the seven-transmembrane receptor Frizzled and the single-membrane-spanning LRP5/6. Frizzled then recruits the intracellular protein Dishevelled leading to the decomposition of the multiprotein β-catenin destruction complex that includes serine/threonine kinases GSK3 and CK1 and tumor suppressors Axin and APC. This results in the accumulation of the active unphosphorylated β-catenin followed by its translocation to the nucleus where it regulates the target gene transcription. The Notch pathway is induced when a delta-like or Jagged ligand binds to the extracellular domain of the Notch transmembrane receptor. This binding causes the proteolytic cleavage of an intracellular fragment NICD which, upon release, localizes to the nucleus and functions to regulate transcription of Notch target genes by interacting with CSL and coregulators. *CSL* CBF1/Suppressor of Hairless/LAG-1, *NICD* Notch intracellular domain, *LRP5/6* Low-density lipoprotein receptor related protein 5/6, *APC* Adenomatous polyposis coli, *TCF/LEF* T-cell factor/lymphoid enhancer factor, *CK1* Casein kinase 1, *SUFU* suppressor of fused protein, and *MAM* Mastermind

Delineating and targeting signaling pathways crucial for the maintenance and epigenetics of PCSCs has gained paramount importance for improving chemotherapeutic outcomes in PC. Similar to their normal tissue stem cell counterparts, PCSCs are regulated by an array of signaling pathways, such as Notch, Hedgehog (Hh), Wnt/β-catenin, NF-κB, PI3K/Akt, JAK/STAT3, and phosphatase and tensin homolog (PTEN). Amongst these, Notch, Hh, and Wnt pathways have been assigned important regulatory tasks in PCSC biology, specifically in PCSC self-renewal, tumorigenicity, invasion, metastasis, and therapy-resistance [157, 158] (Fig. 1).

### Hedgehog pathway

The Hh signaling is a major orchestrator of several fundamental processes in morphogenesis, controlling cell differentiation, cell fate determination, stem cell maintenance and self-renewal, and tissue polarity [159, 160]. Normally this pathway ceases after embryogenesis; however, its aberrant reactivation has been associated with PC invasiveness and tumorigenesis [32]. Hh signaling is initiated by the binding of processed and lipid-modified Hh-ligands, such as Desert Hedgehog (Dhh), Indian Hedgehog (Ihh), and Shh, to their cognate receptors, Patched (Ptch; Ptch1 and to a lesser extent, Ptch2) [159, 161]. CSCs have been shown to respond to Hh ligands by modulating the expression of pluripotency sustaining genes, including Sox2, Nanog, and B cell-specific moloney murine leukemia virus insertion site 1 (BMI1) [159]. In line with this, PCSCs display upregulation of Shh and BMI1 [162]. A number of studies have demonstrated the crucial regulatory role of the Hh signaling pathway in the maintenance of stem-like properties of PCSCs [163, 164]. For example, inhibition of Hh signaling via knockdown of Smoothened (SMO) transmembrane receptor protein, a positive regulator of Hh signaling pathway, inhibited self-renewal, EMT, chemoresistance, tumorigenesis, invasion, and pulmonary metastasis of PCSCs [165]. Huang et al. demonstrated that cyclopamine-mediated inhibition of Hh depressed proliferation and self-renewal of PCSCs via BMI1. Notably, they found that cyclopamine also reversed chemoresistance to GEM by decreasing the expression of ABC transporter protein ABCG2 in PCSCs [166], suggestive of the role of Hh signaling in both self-renewal and reversal of chemoresistance. Inhibition of Hh using GANT61 and cyclopamine was also found to inhibit the sphere formation ability of Capan-1 M9 PC cells [167]. Similarly, inhibition of Shh through baicalein abrogated the self-renewal capability of PCSCs, determined from their reduced sphere formation and reduced colony formation potentials [168]. Additionally, knockdown of Gli protein, a transcriptional effector of the Hh signaling pathway, reduced sphere formation and cell viability of Capan-1 M9 cells. DNA microarray analysis of Capan-1 M9 determined the upregulation of Gli in spheroids, indicating the involvement of the Hh pathway in PCSC self-renewal and maintenance [167]. Considering the contribution of Hh signaling to PCSC properties and chemoresistance as well as tumorigenesis and metastasis of PC, targeting this pathway can only prove beneficial in the treatment of PC.

### Wnt pathway

Classified as an evolutionarily conserved pathway, the canonical Wnt signaling cascade serves crucial roles in both embryonic development and tumorigenesis [11]. The Wnt/β-catenin signaling is one of the classical pathways involved in CSC differentiation, proliferation, and maintenance [169]. Several studies have confirmed the relationship between Wnt-regulated CSCs and the progression of colorectal cancer [170], breast cancer [171], hematologic cancer [172], skin cancer [173], lung cancer [174], and PC [175]. Broeck et al. identified SP in human PDAC resection specimens, typified with the expression of genes involved in chemoresistance and PCSC characteristics as well as the upregulation of the Wnt/β-catenin signaling pathway [176]. Several studies have shown the potential of targeting the Wnt/β-catenin signaling pathway in PCSC subsets to enhance the chemosensitivity of PC cells [177]. In pancreatic xenograft models, treatment with GEM has been shown to increase EMT; however, a combination of GEM with Wnt inhibitor OMP-18R5 resulted in reduced EMT. Additionally, the combined treatment of GEM and OMP-18R5 also caused a reduction in the number of cells exhibiting tumor-initiating properties [178].

### Notch pathway

The highly conserved Notch signaling pathway directs several different developmental and adult tissue homeostatic processes [179]. The core Notch pathway is very simple (Fig. 1); however, the fact that it operates in many different contexts with diverse functional outputs has always been intriguing. Another intriguing aspect is the one-to-one ligand-receptor interaction that is different from the level of regulation standard to many signaling pathways. Several different regulatory mechanisms have been identified that underscore the activity and differing outcomes of the Notch pathway, including the ligand-receptor interaction, the tissue organization, extent of cell–cell contacts, the nuclear environment (cell-type-specific transcription factors and chromatin organization), and the gene regulatory networks in recipient cells [180]. Perturbations in these regulatory mechanisms have been shown to contribute to Notch-related diseases such as cancer. Abnormal activation of the Notch pathway has been detected in the CSCs of breast cancer, glioblastoma, and PC [181].

In the context of PC, Notch pathway plays an important role in maintaining the PCSC population. Quantification of Notch signaling components in CSC and non-CSC populations derived from primary human pancreatic xenografts have shown an upregulation of Notch ligands Notch-1, Notch-3, Jagged (Jag) 1, 2, and Notch target gene HES1 in PCSC subsets. Inhibition of the Notch pathway by a *γ*-Secretase inhibitor (GSI) or HES1 shRNA has been shown to reduce the percentage of ESA + /CD44 + /CD24 + CSCs and suppress their self-renewal and tumorigenicity whereas its activation by delta/Serrate/Lag-2 peptide reverses the suppression [109]. Additionally, inhibition through quinomycin was shown to reduce the expression of CSC markers EpCAM, CD44, DCLK1, and CD24 [108], supporting the significance of Notch pathway activation in PCSC maintenance and function.

In tandem with these embryonic signaling pathways, several other pathways have been proposed to be involved in regulating PCSC activity, such as autophagy, forkhead box protein M1 (FOXM1) signaling, interleukin 8 (IL-8)/CXCR1), NODAL/ACTIVIN signaling pathways [144], and K-ras/JNK axis [157]; however, the significance of these signaling pathways remains elusive.

## Pancreatic cancer stem cell markers and EMT

The classical description of EMT [182] conceptualized this process as a single binary program typically involving the transformation (shift) between mesenchymal or epithelial states. This traditional paradigm has since evolved such that EMT is now considered as a highly plastic, dynamic transitional process covering a spectrum of intermediate “metastable” phases [183]. Accordingly, EMT represents a continuum between epithelial (E), intermediate (EM; also known as hybrid phenotype or “metastable”), and mesenchymal (M) phenotypes. The transitions between these different phenotypes is controlled by the spatiotemporal regulation of many multi-parametric extrinsic and intrinsic factors [184] including transcription factors (Snail1/2, zinc-finger E-box binding (ZEB) 1/2, Twist1, grainyhead-like transcription factor 2 (GRHL2), ovo-like zinc finger (OVOL) 1/2, and paired related homeobox 1 (PRRX1), collectively referred to as ‘EMT-inducing transcription factors’; EMT-TFs), post-transcriptional gene regulators (miRNAs), and the epigenetic regulators [185]. EMT and its intermediate state are integral to several physiologic and pathologic processes such as tissue regeneration, scarring and fibrosis, and cancer development [186–188]. During tumorigenesis, EMT is initiated by a conjunction of environmental changes, evolutionary pressures, and oncogenic events connected to tumor development [189]. Significant parallels between EMT in embryonic development and cancer progression have led to its recognition as a major operator of epithelial-derived malignancies [190, 191], including PC.

A myriad of studies exploring various facets of EMT in PC have been conducted and several reviews written including those directed on delineating the molecular mechanisms of EMT regulation [192, 193], therapy development and resistance [15, 194, 195], and metastasis [196]. Studies in the last decade have focused on another aspect - the EMT-CSC link, fueling interest in deciphering the contribution of EMT to CSC marker expression, self-renewal, clonogenicity, and tumorigenicity of PC cells.

The formulation of stochastic and the hierarchal CSC models, crediting phenotypic plasticity of cancer cells (that is, transient and reversible transformations between CSC and non-CSC traits) to tumor formation and progression [197], has greatly enhanced our understanding of the major epigenetic mechanisms or “tags” [198] that control the phenotypic diversity of distinct tumor cell subpopulations within a tumor mass. Specifically, EMT has been shown to impart heritable morphological and physiological changes to carcinoma cells without concomitant changes in their nucleotide sequences/genomes [15], notably disruption of epithelial cell–cell junctions, conversion from apico-basal polarity to front-rear polarity, gain of mesenchymal traits (marked by N-cadherin, vimentin, α-smooth muscle actin (SMA), and fibronectin) and loss of epithelial markers (E-cadherin, γ-catenin, and zonula occludens-1 (ZO-1)), remodeling of junctional complexes to favor cell-substrate adhesions [199], acquisition of motility and invasion, and restructuring the expression status of a minimum of 400 distinct genes (termed ‘EMT cancer signature’) [12, 142]. In several carcinomas, only the tumor cell subpopulation that is enriched in CSCs exhibit these traits associated with this “canonical” program [200, 201]. Meanwhile, EMT also induces the expression of stem cell markers, suggesting the mutually exclusive relationship between EMT and molecular and functional stem cell traits [202].

Since EMT is orchestrated by one or many classical EMT-TFs often associated with features of stemness, it is not surprising that CSC-enrichment is seen in tumors with high expression of EMT-TFs [203]. Reports have outlined the engagement of CD44 in the EMT gene regulation and the activation of an invasive program in PC. Jiang et al. revealed that activation of Snail1 upon CD44 overexpression induces a mesenchymal phenotype and regulates the invasive capabilities of the PC cells via membrane-bound metalloproteinase (MMP-14/MT1-MMP) expression, thus establishing the key regulatory effect of CD44-Snail-MMP axis in the EMT program and invasion in PC [110]. Activation of Snail1 is implied in disrupting the asymmetric stem cell division leading to hyperproliferation and stem cell expansion [204]. Another EMT-TF engaged by CD44 is ZEB1. ZEB1 is significantly associated with poorly differentiated pancreatic tumors and can suppress the expression of stemness-inhibiting miR-200 family members and miR-203, resulting in the induction of the EMT program and maintenance of stemness [205]. miRNAs constitute one of the upstream regulatory mechanisms controlling the expression and functions of EMT-TFs [206]. Amongst the best-characterized miRNAs regulating the EMT program, miR-200 family members are implied in attenuating the expression of ZEB1 and ZEB2 and (intriguingly) vice-versa, thus forming a double-negative regulatory feedback loop [207, 208]. In PC, ZEB1 enforces alternative splicing of variant CD44v isoform to the standard CD44s isoform by repressing epithelial splicing regulatory protein 1 (ESRP1) [111]. CD44s, in turn, upregulates ZEB1 expression, resulting in a self-enforcing feedback loop with a functional impact on tumorsphere-forming capacity, drug resistance, and tumor recurrence. Research has highlighted the functional role of ZEB1-mediated EMT in MDR, providing a rationale to inhibit ZEB1 which in turn would mitigate EMT features in PC [209]. Subsequent studies revolving around ZEB1-mediated EMT have also demonstrated its role in the acquisition of CSC-like phenotype in GEM-resistant PC cells via the activation of Notch signaling [210].

A linear relationship between CD133 expression, invasion, drug resistance, and EMT has been described [43]. CD133 overexpression in MIA PaCa-2 cells increases cellular invasiveness ​mediated by a significant upregulation in EMT-TFs (Snail1 and ZEB1) and other EMT-associated markers (vimentin, N-cadherin, MMP-9) [43]. CD133 imparts a critical role in facilitating the EMT regulatory loop; in CD133 + highly migratory PC cell line, Capan-1 M9, CD133/Src/Slug signaling axis upregulates N-cadherin expression facilitating invasion and metastasis of PC cells [113]. Under unfavorable hypoxic conditions, CD133 confers tumorigenic potential and survival advantage to PCSCs via EMT, particularly through upregulation of Slug and N-cadherin levels [114]. In addition, Slug has been shown to impart GEM-resistance to CD133 + PCSCs through EMT [112].

Overexpression of PCSC marker nestin accounts for increased cell motility and EMT-associated phenotypic changes *in vitro* [211]. Nestin is a cytoskeletal intermediate filament protein that participates in maintaining cell integrity, migration, and differentiation [211, 212]. Originally classified as a functional neuroepithelial stem cell protein in developing and adult brains [213], nestin is now used to characterize stem or progenitor cells and CSCs in pancreatic, brain, ovarian, head and neck, and prostate tumors [214–216]. Compared with parental cells, nestin-expressing metastatic PDAC cells display EMT and CSC features that are induced via the nestin-mediated increase of Slug [217]. Reports have also suggested the interaction between nestin and another EMT-TF, Snail; endogenous nestin bestows increased migratory, invasive, and metastatic abilities to PDAC cells by upregulating Snail and repressing E-cadherin [218]. Nestin expression status in epithelial cell types is proposed to be regulated by two pivotal factors—hypoxia and transforming growth factor β (TGF-β) [219]. EMT and CSCs share key biological characteristics, such as resistance to cytotoxic T lymphocytes (CTLs) and reliance on TGF-β signaling [189]. Su et al. uncovered a positive cross-regulatory loop between nestin-TGF-β1/Smad and EMT in PDAC following a hypoxic stimulus [211]. Overexpression of nestin in MIA PaCa-2 (Smad4-proficient) cells was shown to induce a Smad4-dependent upregulation of TGF-β1 as well as enhance the expression levels of TβR receptors that further support an autocrine TGF-β1 signaling cascade. This activated TGF-β1/Smad signal, coupled with nestin protein expression, induced EMT, typified by the downregulated expression of E-cadherin and the upregulated expression of vimentin, N-cadherin, and SMA. Mouse xenograft studies have supplemented the role of nestin in promoting autonomous PDAC tumor metastasis through autologous activation of TGF-β1/Smad signaling [211].

Reports indicate that EMT activation is probably one of the mechanisms that underscore the involvement of DCLK1 in PC metastasis [121]. Accordingly, a correlation between DCLK1 + CSCs, EMT, angiogenesis, and immune checkpoint has been proposed. As observed in pancreatic tumor xenograft models, siRNA-mediated knockdown of DCLK1 or downregulation by a kinase inhibitor XMD8-92 leads to the decreased expression of angiogenic markers/vascular endothelial growth factor (VEGF) receptors (VEGFR1 and VEGFR2) and EMT-TFs ZEB1, ZEB2, Snail, and Slug [120, 122]. In compliance with these observations, siRNA-mediated knockdown of DCLK1 was shown to decrease BMI1, Snail, and vimentin expression and enhance E-cadherin expression in both PC cell lines and xenografts in nude mice [121]. Expectedly, a significant correlation of DCLK1 expression with BMI1, Snail, and vimentin attributes to the mesenchymal features and increases proliferation in clinical samples. Accumulating evidence demonstrates that BMI1, a key polycomb group protein, governs stem self-renewal and promotes malignant transformation [220, 221] via EMT and downregulation of E-cadherin in PC. DCLK1 has also been shown to elevate programmed cell death ligand 1 (PD-L1) expression and regulate CTL infiltration via miRNA-200/ZEB1 axis [222] and by affecting the yes-associated protein (YAP) expression in the Hippo pathway [119]. It is speculated that PD-L1 helps DCLK1 + CSCs to avoid immune surveillance, thus contributing to the expansion of immunosuppressive TME. Moreover, this process might be connected to DCLK1 regulatory activity on EMT, considering EMT is associated with immune checkpoint during tumor development [223]. Cumulatively, DCLK1 + CSCs and EMT represent a tandem target of therapeutic intervention, particularly, checkpoint blockade therapies, against metastatic PC.

CD24 is well accepted as a CSC marker, but results on its contribution to EMT are contradictory. While tumor cells undergo EMT, some studies have determined downregulation (as seen during TGF-β-dependent EMT) [29], while others have observed upregulation of CD24 expression [224]. The CD44 + CD24 + CSC populations derived from PC cells exhibit signs of EMT program activation, including mesenchymal phenotype related to increased vimentin, and reduced E-cadherin levels [224]. Intriguingly, there have been reports on the inhibition of metastatic gene signatures, downregulation of Twist, and upregulation of β-catenin expression (via crosstalk with the Wnt/β-catenin pathway) correlating to the CD24 expression in tumor cells [29]. Although CD24 expression regulates both epithelial and mesenchymal markers, surface CD24 has been shown to stabilize an epithelial phenotype during PC development and generate differentiated tumors marked by strong β-catenin expression and absence of Twist expression [29]. Future studies are required to gain mechanistic insights into the factors that guide the distribution, localization, and translocation of CD24 in the intracellular compartments. Also, efforts should be directed at uncovering new elements that are fundamental to the role of CD24 in the dynamics of EMT in PC.

Despite the substantial proof linking EMT and CSC state, recent studies have provided conflicting evidence on uncoupling EMT and stemness and the existence of a parallel non-redundant EMT pathway [183, 225]. While EMT-program activation in the otherwise-epithelial carcinoma cells is essential for distant metastasis, full EMT may prove detrimental to tumorigenic activity by locking cells in fully differentiated states and diminishing their plasticity [225, 226]. Therefore, as an alternative to full EMT, most cancer cells undergo phenotypic drift against environmental stimuli, termed intermediate or hybrid EMT, that support their adaptation and survival. Hybrid, reversible transitions confer both epithelial and mesenchymal characteristics to cells, potentially endowing them with more migratory capabilities [227] while manifesting a high degree of plasticity and increasing their susceptibility to acquire stemness [183, 228]. This intermediate state is noted in PC, whereby circulating tumor cells (CTCs) exhibit stem cell properties but with low expression levels of E-cadherin and simultaneous mesenchymal features [229]. It has also been proposed to underscore the presence of “migratory CSCs” at the invasive borders of tumors [230]; in PC this “migrating” and a highly metastatic population is characterized by cells co-expressing CD133 and CXCR4 [23].

Evidently, cancer cells can exhibit distinct EMT states which generate vastly different phenotype readouts and confer high levels of plasticity to enable the formation of macrometastasis at distant fertile sites. Cumulatively, the discovery of the EMT-CSC link has made a major contribution to the oncogenic PC network. Still, extensive research is warranted to eliminate existing ambiguities and open new diagnostic and therapeutic avenues for PC.

## Clinical manifestations of pancreatic cancer stem cells

**Fig. 2:**
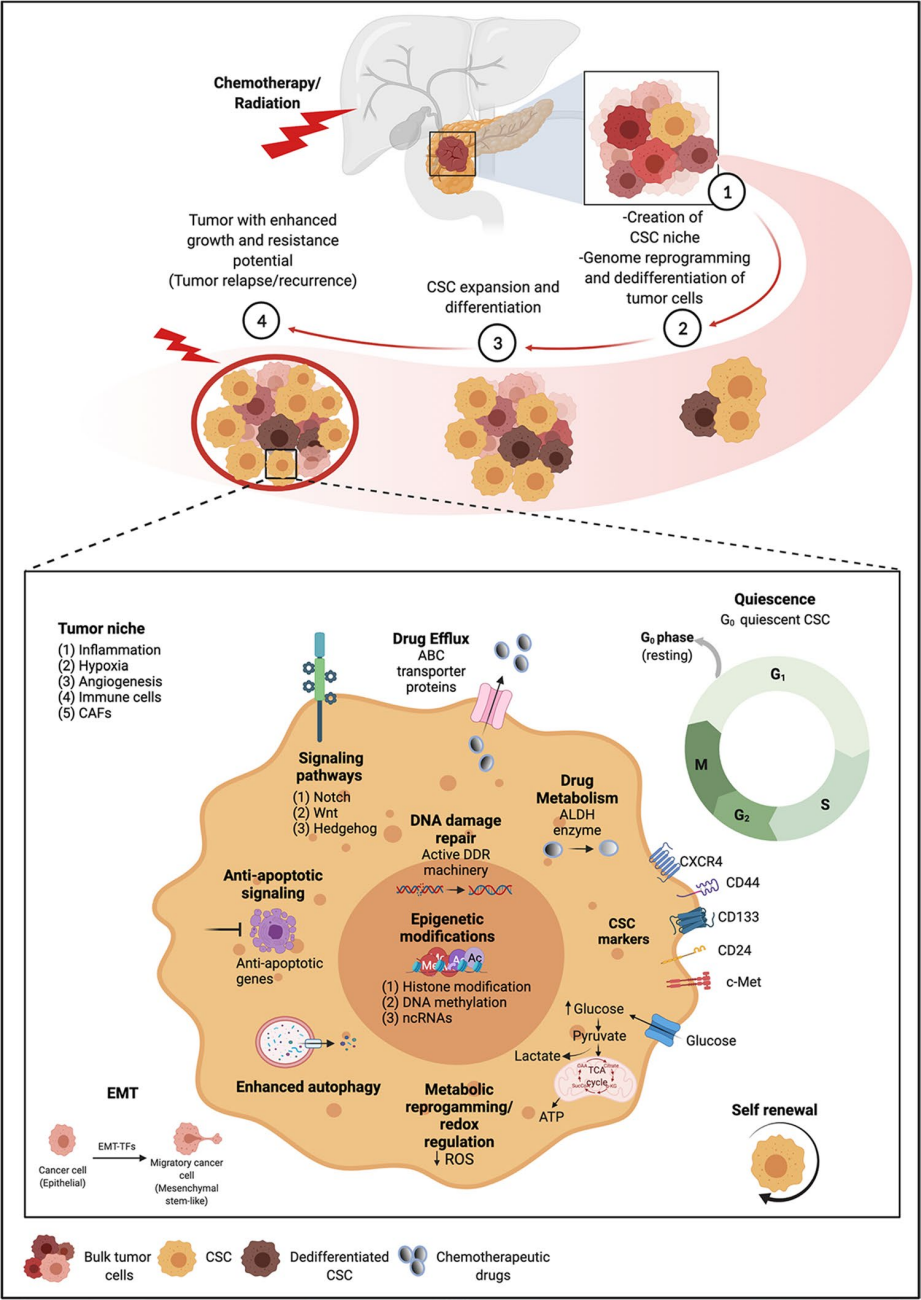
CSC-mediated mechanisms underscoring therapeutic resistance in pancreatic cancer. Multiple intrinsic and extrinsic mechanisms induce the chemoresistant phenotype in PCSCs. When a tumor is exposed to systemic chemotherapy and/or loco-regional radiation therapy, the majority of the bulk tumor cells get eradicated but not the CSCs. In due course, a CSC niche is created that favors the stemness potential and activity in CSCs. The oncogenic insults also favor the bidirectional conversion between CSCs and non-CSCs; tumor cells undergo genome reprogramming and dedifferentiate to a progenitor/stem cell state and create a new pool of CSCs. Eventually, these therapy-resistant CSCs expand and repopulate the tumor and generate additional therapy-resistant CSC progeny. This tumor plasticity leads to tumor relapse and recurrence. During treatment or post-therapy, PCSCs display several features such as improved DNA repair capacity, a higher degree of drug efflux activity, increased metabolic reprogramming, quiescence, EMT, enhanced autophagy, epigenetic modifications, tumor microenvironmental interactions, and dysregulated developmental pathways that all enable them to stay resilient within a tumor, evade anti-proliferative therapies, and recur in post-therapy cancer patients

The fact that therapy-resistance is driven by pre-existing or therapy-induced chemoresistant clones has prompted a better understanding of CSC features and selectively identifying their peculiarities for developing efficient therapeutic rationales in cancer. Evidently, CSCs harbor innate resistance to chemotherapy and radiation, attributed to the dysregulated developmental pathways, EMT, and cell surface markers. Beyond these, CSC-mediated chemoresistance is majorly governed by improved DNA repair capacity, increased tolerance to DNA damage, elevated levels of detoxification enzymes, quiescence, epigenetic modifications, and the tumor microenvironmental interactions and pressures [231, 232] (Fig. 2).

### Therapeutic-resisting mechanisms employed by pancreatic cancer stem cells

#### Quiescent CSCs evade chemotherapy-induced damage

The quiescent nature of CSCs represents a mechanism by which CSCs stay resilient within a tumor, evade conventional anti-proliferative therapies, and recur in post-therapy cancer patients. Experimental evidence generated from both in vitro and in vivo studies has confirmed the existence of a subpopulation of slow-cycling tumor cells with a capacity to survive chemotherapeutic treatment when compared to bulk tumor cells [233]. Such a population of slow-cycling cells (DiI + /SCC) fulfilling the operative criteria of CSCs has been identified in PC cell lines [233]. These slow-cycling stem cell-like subpopulations manifest a panel of tumor-related alterations such as EMT-mediated increase in invasiveness and tumorigenic potential, ability to reproduce heterogeneous tumor cell population, upregulation of the Hh/TGF-β pathway, partial overlap with the CSC markers CD24/CD44, CD133, and ALDH and evasion of chemotherapy-induced death stimuli [233]. Investigations have also identified a subpopulation of dormant PC cells resistant to the genetic and pharmacologic ablation of oncogenic pathways, exhibiting CSC-like features and relying on mitochondrial oxidative phosphorylation (OXPHOS) for survival [234]. Concomitantly, de-differentiated PC cells have been shown to progressively increase the expression of stem cell markers, undergo EMT, switch from glycolysis to oxidative metabolism, and, eventually, gain a slow-cycling/quiescent stem state with a global metabolic shut-down [235]. This acquisition of quiescent stem cell state, characterized by high chemoresistance, clonogenic ability, and metastatic potential, is likely to occur in response to different tissue oxygen tension (particularly hypoxia) [81], lack of nutrients, detachment from the substratum [236], or even chemotherapy-induced damage [237]. Redifferentiation via mesenchymal-to-epithelial transition (MET) may reawaken these dormant cells into a highly aggressive phenotype by re-activating their proliferative capacity and glycolytic metabolism [235]. Undoubtedly, quiescent CSCs represent major targets of therapeutic strategies designed to eradicate and/or prevent a lethal metastatic recurrence.

#### PCSCs respond to genotoxic stress via active DDR machinery and metabolic reprogramming

Radiation and numerous anti-cancer drugs such as DNA-reactive agents (Cisplatin, Oxaliplatin, and Carboplatin), inhibitors of nucleotide metabolism pathways (5-FU, Capecitabine, Floxuridine, GEM, Mercaptopurine, 8-Azaguanine, Fludarabine, and Cladribine), anti-metabolites inhibiting DNA synthesis (Methotrexate), and topoisomerase poisons (Doxorubicin and Daunorubicin) have been identified as effective mediators of DNA damage and inducers of cancer cell death [238]. Cancer cells typically exhibit relaxed DNA damage repair capabilities and high proliferative potential owing to their capacity to ignore cell cycle checkpoints. Although beneficial, these capacities also make cancer cells more susceptible to DNA damage and, hence, cell death [238]. Accruing evidence has appreciated the activation of the DNA damage sensor and repair machinery as one of the protective mechanisms adopted by CSCs to overcome chemotherapy- and radiotherapy-induced damages [239]. While a proficient DNA damage repair (DDR) machinery helps in safeguarding genomic integrity following endogenous and exogenous insults [239], CSCs have been shown to aberrantly activate the repair pathways and maintain a superior DNA repair profile as against the bulk tumor cells to bypass chemotherapeutic damage [240]. The correlation between DDR signals and CSCs chemoresistance in PC stems from the significant increase in the expression of cell cycle- and DDR-related genes, particularly breast cancer type 1 susceptibility protein (BRCA1), observed in PCSCs following treatment with GEM [240]. Both inherited and sporadic human PDACs harbor somatic or germline mutations in DDR genes, such as BRCA1/2, partner and localizer of BRCA2 gene (PALB2), and ataxia telangiectasia mutated (ATM) [241], and therefore, efforts are directed towards developing effective DDR inhibitors or identifying various components of the DDR machinery amenable to inhibition. Moreover, given a strong relationship between cell cycle and DDR, quiescent CSCs exhibiting slow replication kinetics adopt error-prone low-fidelity nonhomologous end-joining (NHEJ) pathway when damaged, thereby generating a new mutation that is passed onto the progeny. This transmission endows the progeny with an enhanced metastatic ability or increased chemoresistance [239]. Hence, considering the potential contribution of quiescence to both chemoresistance and genetic instability in CSCs, the combinatorial use of inhibitors targeting quiescence-associated signaling pathways and DDR effectors may prove efficacious in the eradication of CSCs.

Additional mechanisms implicated in CSC-driven resistance to genotoxic stress are related to efficient scavenging of radiation-induced free radicals, including ROS as well as lower ROS levels than corresponding non-CSC populations [242]. Given the critical role of ROS toxicity in irradiation-induced cell death [242], CSCs, with lower ROS levels and enhanced ROS defenses, develop less DNA damage and radioresistance in comparison to their non-tumorigenic progeny [242]. In addition to radioresistance, evidence has linked low ROS levels with stemness and EMT properties in CSCs [242, 243]. Moreover, ROS has also been linked to energy metabolism plasticity in CSCs [244], making them readily switch their metabolic state depending on the energy requirements [245]. Similar to the phenotypic heterogeneity, CSCs harbor complex metabolic profiles relative to the bulk of the tumor [246]. In line with this, Sancho et al. identified metabolic heterogeneity within PCSCs; CD133 + PCSCs predominantly rely on OXPHOS and possess a very limited metabolic plasticity. However, treatment with anti-diabetic drug metformin results in the emergence and expansion of resistant, metabolically plastic CSC clones with an intermediate glycolytic/respiratory phenotype, suggestive of a metabolic switch in the OXPHOS-dependent population [46]. Consistent with these findings, Zhao et al. demonstrated that GEM-resistant PC cells exhibit greater glycolysis flux than their parental cells, and this enhanced glycolytic signaling regulates the CSC and EMT phenotypes via lowering ROS production and increasing DCLK1 expression [247]. Cancer cells have been shown to reprogram their metabolic circuitry, in particular during EMT, to meet the increased bioenergetic demands following diverse metabolic challenges [248, 249]. In recent years, several studies have focused on metabolic rewiring that occurs during EMT in an effort to identify key metabolic nodes vulnerable to therapeutic targeting. Metabolic reprogramming via glycolysis is a known contributor of EMT, as evident from the functional role of glycolysis-ROS-DCLK1 pathway in the development of GEM-resistance and acquisition of EMT/CSC features in PC [247]. Considering this novel ROS-mediated metabolism/stemness perspective in chemoresistant PC, a combinatorial strategy involving inhibition of glycolysis, knockdown of DCLK1, and upregulation of ROS has been proposed to enhance chemosensitivity in PC. Alongside glycolysis, PCSCs also utilize the non-canonical pathway of glutamine metabolism to maintain redox balance and low ROS levels [250]. Glutamine deprivation or inhibition of glutamic-oxaloacetic transaminase sensitizes PCSCs to fractionated radiation in vitro and in nude mice via enhanced intracellular ROS generation [250]. All these findings substantiate the fact that even within the same tumor, different CSC subpopulations harness different metabolic strategies and metabolic potentials under stressed conditions. In view of the functional heterogeneity of CSCs within PC, a marker-independent approach to study the properties and vulnerabilities of CSCs has been proposed. Accordingly, Domenichini et al. have proposed the reliability of PC tumorspheres, owing to their unique metabolic profiles, as a novel predictive in vitro model to identify and analyze CSCs, test chemoresistance, and validate new metabolic vulnerabilities in PC [246].

#### PCSCs increase the activity of detoxification enzymes

Concurrent studies analyzing the mechanisms of MDR phenotype in PC have demonstrated its strong association with the overexpression of detoxifying enzymes, together with certain drug efflux transporter proteins [250, 251]. CSCs have been intimately related to drug resistance in PDAC due to the overexpression of detoxifying enzymes such as ALDH that are involved in cellular drug metabolism. In fact, high ALDH activity is associated with putative CSC populations in human PC exhibiting enhanced tumorigenic potential [251, 252]. Amongst the numerous enzyme isotypes, ALDH1A1 is the key ALDH isozyme that is linked to CSC function in cancers, particularly, self-renewal, differentiation, and self-protection [253]. In PC, GEM-resistant cells show significantly higher expression and activity of endogenous ALDH1A1 in comparison to parental cells that account for both *de novo* and acquired resistance to GEM [254]. Knockdown of ALDH1A1 markedly inhibited cell proliferation and increased sensitivity to GEM, indicating a vital functional role of ALDH1A1 in maintaining drug resistance in tumor cells. Moreover, treatment of GEM-resistant PDAC cells with the combination of ALDH1A1-siRNA and GEM significantly decreased cell viability, increased apoptosis, and induced cell cycle arrest at the S-phase, thereby proving the potential of this combinatorial treatment to repress GEM-resistance in PC [254].

#### PCSCs employ epigenetic mechanisms to combat clinical intervention

Advances in genome-wide technologies have widened our knowledge on the epigenome dynamics and the interplay between epigenetic marks during different cell state transitions, such as stem cell differentiation and lineage commitment in adult tissues [255]. Recent findings have shed light on the dynamics and involvement of key epigenetic regulatory events in shaping the transcriptional landscape of embryonic stem cells [255]. Accumulating evidence has now identified epigenetic alterations and the reorganization of epigenetic signatures as the potential mechanisms in shaping transcriptional dysregulation that occurs during CSC formation and maintenance [256]. Epigenetic reprogramming via DNA methylation, histone modifications, and noncoding RNAs (ncRNAs) such as miRNAs, lncRNAs, and circular RNAs (circRNAs) has been demonstrated to regulate CSCs that participate in the etiology and progression of various cancers, including PC.

The epigenetic regulation of chemoresistance has been extensively studied in CSCs addressing dysregulation/perturbations of microenvironmental interactions, classic CSC signaling pathways, and the gene expression profiles related to cell proliferation, metabolism, and survival [257]. With the identification of ncRNAs as important epigenetic regulators, there has been an explosion of studies focusing on the role of these RNA transcripts in regulating recurrence and metastasis of malignancies, including CSC-driven therapy-resistance. miRNAs are a kind of small ncRNAs (sncRNAs; 19–24 nt) that are well-known for their pleiotropic effects on the signaling cascades, physiological phenomena, and cellular properties [258]. By the RNA-splicing mechanism, miRNAs target multiple mRNAs related to oncogenesis (oncomiRs), progression, and metastasis (metastamiRs), as well as MDR (MDRmiRs) [259]. A number of dysregulated miRNAs have been associated with drug resistance of PC [260] and reportedly studied in the bulk of tumors. An array of differentially expressed miRNAs (such as miR-99a, miR-100, miR-125b, miR-192, and miR-429) and mRNAs have been detected in PCSCs [261]; still, very few reports have provided evidence on the direct contribution of cancer stemness-associated miRNAs in PC chemoresistance. For example, Hasegawa et al. demonstrated the stimulatory role of miR-1246 in inducing GEM-resistance and CSC-like properties both in vitro and in vivo via targeting cyclin G2 (CCNG2), a tumor suppressor gene [262]. Instead, a plethora of studies have established a strong correlation between tumor suppressor miRNAs and PCSC-driven drug resistance and determined how their modulation (replenishment/re-expression) helps in restoring chemosensitivity to GEM. Singh et al. detected differential expression of miRNAs in GEM-resistant MIA PaCa-2 cancer cells and clinical metastatic PC tissues [263]. The authors identified a set of miRNAs that were either upregulated (such as miR-146) or downregulated (such as miRNA-205, miRNA-7) in the PC cells analyzed. miR-205 functions as a tumor suppressor miRNA and hence, one of the most downregulated RNA transcripts in a variety of cancers, such as malignant melanoma [264], prostate cancer [265], and head and neck squamous carcinoma [266]. Functionally, miRNA-205 replenishment was shown to restore chemosensitivity to GEM in MIA PaCa-2 cells by decreasing the expression of stem cell markers Oct3/4 and CD44 in ALDH-positive CSC fraction as well as targeting class III β-tubulin (TUBB3), a predictive marker for GEM/NabP resistance in PC [263, 267]. Similarly, miR-17–92 cluster has been identified as one of the functionally defining epigenetic signatures in GEM-resistant PCSCs, overexpression of which can counteract stemness and GEM-resistance via reduced CSC self-renewal capacity, targeting NODAL/ACTIVIN/TGF-β1 signaling cascade as well as directly inhibiting its downstream targets p57, p21, and T-box transcription factor 3 (TBX3) [268]. Loss of another miRNA, miR-34, has been detected in CD44 + /CD133 + tumorsphere-forming and tumor-initiating PCSCs, accompanied with increased levels of Notch/Bcl-2. Functional restoration of miR-34, a bona fide tumor suppressor and downstream target of p53, in human p53-mutant PaCa2 cells resulted in the downregulation of Bcl-2 and Notch-1/2, accompanied by significant inhibition of clonogenic cell growth and invasion, increased apoptosis and cell cycle arrest, and augmented sensitivity to chemotherapy and radiation [269].

It is increasingly clear that EMT-type cells and CSCs are potent effectors of tumor relapse and chemoresistance. Given the ubiquitous regulatory roles of miRNAs in EMT and CSCs, attention has now been focused on the identification of lncRNAs that control the EMT and CSC phenotypes as well as GEM-resistance in PC, partly, through their regulatory function with miRNAs [270]. To this vein, linc-DYNC2H1-4, a long intergenic ncRNA (lincRNA; a class of autonomously transcribed RNAs that do not overlap protein-coding genes) [271] has been shown to regulate EMT and CSC properties via sponging the tumor suppressor miR-145 in GEM-resistant PC cells [270]. Next-generation sequencing technology has helped identify novel lncRNA signatures in PDAC samples compared to normal tissues [272]. Metastasis-associated lung adenocarcinoma transcript 1 (MALAT-1), downregulated in CSCs, has been shown to enhance the PCSC fraction, promote self-renewal via Sox2, confer GEM-resistance, accelerate tumor angiogenesis in vitro, and promote PC cell tumorigenicity in vivo [273]. MALAT1 acts as a competing endogenous RNA (ceRNA) for both miR-200c and miR-145 that targets stemness gene Sox2 [273, 274]. It is speculated that MALAT1 regulates PCSCs via the miR-200c/miR-145/Sox2 signaling axis [275]. Similarly, HOX antisense intergenic RNA HOTAIR, enriched in PCSC population following exposure to GEM, was demonstrated to augment the self-renewal capacity, proliferation, and migration of the PCSCs. Interestingly, lentivirus-mediated introduction of HOTAIR and not GEM-induced expression promoted resistance to GEM and the stem-like phenotype in PC cells [276]. Emerging data has also presented the involvement of tumor suppressor lncRNAs in regulating chemosensitivity to GEM in PC cells. For instance, Ma et al. uncovered the functional role of maternally expressed gene 3 (MEG3) as a tumor suppressor and inhibitor of cell proliferation, migration and invasion, EMT, CSC features, and chemosensitivity in PC cells [277]. Despite a myriad of investigations focused on the modulatory role of epigenetic mechanisms in drug resistance in PC, research on the epigenetic landscape and its impact on chemoresistance in PCSCs is still at the nascent stage. The available reports on epigenetic modifications in PCSC chemoresistance are limited to only a few classic drugs and mainly focused on miRNAs. Therefore, it is necessary to systematically address molecular mechanisms underlying the influence of various epigenetic modulators in shaping the identity, generation, and development of drug resistance in PCSCs for further comprehension of their role as biomarkers and therapeutic targets of PC.

#### Bidirectional communication between PCSCs and the components of the TME confers therapy-resistance

It is widely accepted that TME can temporally and spatially regulate the interplay between tumor cells and CSCs which is intricately controlled by the cues in the form of secreted factors and cell–cell contacts. This dynamic cross-talk encompasses the communication between CSCs, non-CSCs, and tumor stromal cells. Through the communication loop with tumor stromal cells, CSCs can self-regulate as well as regulate the TME and processes of hypoxia, angiogenesis, metastasis, and immune evasion [278]. Accumulating evidence suggests that, within or adjacent to the TME, CSCs reside in a tightly controlled anatomically specialized regions, referred to as the “CSC niche.” This niche essentially regulates CSC fate and divisional dynamics by the virtue of signaling cues derived from secreted factors or via cell-to-cell contacts [231, 279]. Cells within the CSC niche stimulate various signaling pathways [280], particularly Notch [281], and Wnt [282] pathways, enabling CSCs to metastasize, evade anoikis, and undergo symmetric division [283, 284]. It is noteworthy that considerable differences exist between the TME and the CSC niche even in the same cancer type or subtype, adding to our incomplete understanding of these distinct microenvironments.

**Fig. 3:**
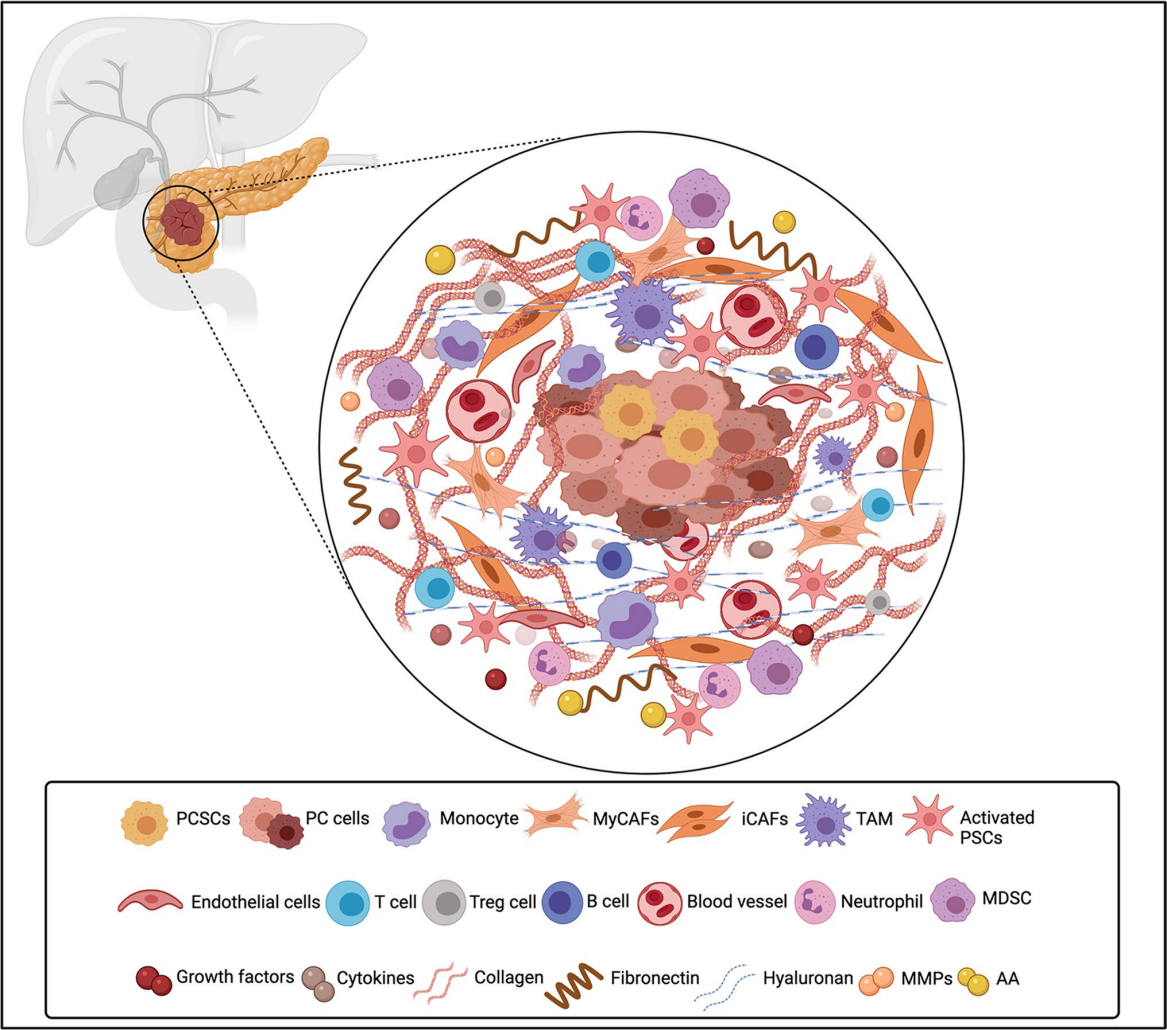
The primary tumor microenvironment in pancreatic cancer—emphasis on pancreatic stellate cells. The pancreatic TME is characterized by dense desmoplastic stroma that is majorly occupied by PSCs (nearly 50%). Upon activation by inflammatory signals such as TGF-β1, PSCs present myofibroblast-like phenotype, recruit immunosuppressive cells (MDSCs, TAMs, and Treg cells), and secrete ECM components (collagen, laminin, fibronectin, and HA), inflammatory cytokines (IL-6 and tumor necrosis factor (TNF)-ɑ), pro-angiogenic factors (VEGF), matrix metalloproteinases (MMP-2,9), growth factors (platelet-derived growth factor (PDGF)), and non-essential amino acids (alanine, aspartate). Two major subtypes of CAF have been identified within the tumor stroma, iCAFs and myCAFs. This complexity of the pancreatic tumor TME fosters rapid growth, enhances invasive and metastatic potentials, confers a survival advantage in hypoxic and low-nutrient conditions, and bestows therapy-resistance capabilities in PCSCs and PC cells. *iCAFs* inflammatory cancer-associated fibroblasts, *myCAFs* myofibroblastic cancer-associated fibroblasts, *Treg cell* regulatory T cell, *MDSC* myeloid-derived suppressor cells, and *AA* amino acids

Nevertheless, the TME, particularly PC-associated TME (Fig. 3), has been extensively characterized [285, 286] and studied in the context of its regulation on CSC-ness and plasticity [279]. The PC TME is pathologically characterized by an extensive fibrotic response (also called desmoplasia) that generates dense collagenous and hypoxic stroma [287]. In fact, this abundant stroma represents one of the two hallmarks of PC (the other being poor vascularization) and poses a major challenge in effectively targeting pancreatic tumors [288]. At the cellular level, the PC TME or specifically the tumor stroma is composed majorly of PSCs along with immune cells, inflammatory cells, endothelial cells, ECM, neuronal cells/nerve fibers, BMDCs, and soluble proteins such as growth factors and cytokines [279]. While each of these components influences the functional properties of cancer cells and contributes to chemo- and radiotherapy resistance, PSCs construct a paracrine niche for PCSCs and promote their self-renewal, tumorigenic, chemoresistant, and invasive potentials [288]. In agreement with this, Lonardo and group demonstrated that PSCs promote PCSC phenotype through the paracrine NODAL/ACTIVIN/activin-like kinase (Alk) 4 signaling. Of importance, knockdown of the common NODAL/ACTIVIN receptors Alk4/7 considerably inhibited CSC self-renewal, abolished in vivo tumorigenicity, and blunted GEM-resistance in orthotopically engrafted PCSCs [144]. PSCs have also been shown to enhance the CSC phenotype, EMT, and radioresistance of PC cells via paracrine TGF-β1 signaling [289]. These findings suggest that both stroma and its functionally active components, such as PSCs, confer advantageous chemoresistant properties to PC cells and PCSCs.

Infiltrating immune cells in the PC TME also promotes CSC traits and therapy resistance. Tumor-associated macrophages (TAMs) are the major inflammatory/immune cell infiltrates in PC tumors [290] and the CSC niche [291]. TAMs provide a unique microenvironment and pivotal signals to promote CSC phenotype and functions; in turn, CSCs convey pro-tumorigenic cues to TAMs augmenting tumorigenesis [291]. TAMs have been shown to directly induce CSC state in PC cells by activating STAT3. In return, CSCs facilitate TAM-induced immunosuppression by blocking antitumor CD8 + T lymphocyte responses during chemotherapeutic treatment [292].

Besides the intrinsic pool of resistant CSCs within a tumor, CAFs contribute to the treatment-induced enrichment of CSCs by secreting a plethora of paracrine factors (chemokines and cytokines) implicated in CSC maintenance and/or expansion [293]. In desmoplastic cancers such as PC, CAFs display phenotypic, functional, and genetic heterogeneity that is dynamically controlled by their microenvironments and origin [294]. Following the chemotherapy-induced phenotypic and functional alterations, such as persistent STAT-1 and NF-κB activity, pancreatic CAFs secrete ELR amino acid motif-positive (ELR +) CXCL chemokines, which on binding to cancer cells via CXCR2 actuate their transdifferentiation into the CSC phenotype and promote post-therapy aggressive and invasive behavior [293].

In pancreatic tumors, the ECM-rich stroma acts as a physical barrier for efficient drug delivery, thus contributing to therapy-resistance. In addition, this glycoprotein- and proteoglycan (PG)-rich part of the TME serves as a favorable niche for the enrichment of the treatment-refractory CSC population and caters to their metabolic needs [295]. Several studies have revealed a functional role of members of the glypican family, one of the two major heparan sulfate PG families, in CSCs [295]. For example, glypican-4 (GPC4) was recently shown to regulate 5-FU resistance and PC stemness via stimulating the Wnt/β-catenin pathway [296]. HA, a major ECM component of the PC TME, and CD44 interactions elicit diverse signals that regulate CSC self-renewal, maintenance, and MDR [297] in head and neck [298] and breast [299] cancers; however, the role of HA-CD44 binding in the context of chemoresistance in PCSCs remains unclear.

Collectively, various cellular and acellular components play a key role in maintaining the dynamic equilibrium between CSCs and their microenvironment and conferring beneficial traits for survival. Detailed understanding of the CSC niche will certainly impact therapeutic approaches.

### Clinical perspective: targeting pancreatic cancer stem cells by phytochemicals

Over the last decades, CSCs have garnered great interest owing to their imperative role in virtually all facets of tumor biology, thus making them an attractive target for therapeutic interventions. In PC, mounting evidence has envisaged the potential of targeting PCSCs that can help address cancer regression and prevent relapse of pancreatic malignancies after treatment with therapeutic modalities [11, 300]. Indeed, a number of manipulative strategies have been formulated to target PCSCs to manage tumor progression. In recent times, alternative medicine utilizing “natural agents,” principally phytochemicals, has gained immense attention for their potential therapeutic applications against many cancers including PC [301, 302]. These phytochemicals exhibit diverse pharmacological properties and have intriguing advantages over synthetic chemotherapeutic drugs, mostly, ascribed to their broad safety profiles [303]. Growing evidence indicates that natural compounds exert their therapeutic impact through multidimensional targeting of aberrantly activated cellular and molecular signaling pathways in CSCs [301]. Various preclinical and clinical studies have asserted the role of natural bioactive compounds (isolated dietary phytochemicals or plant-based functional foods) in targeting CSCs, especially in PC [304].

#### Resveratol

Resveratrol (RSV; trans-3,4′,5-trihydroxystilbene) is a polyphenolic phytoalexin widely distributed in grapes, berries, peanuts, and hellebore [305]. RSV modulates a myriad of pathways that accounts for its potent anti-cancer effects including induction of apoptosis, increased radiosensitivity, cell cycle arrest, decreased cell proliferation, inhibition of invasion/metastasis, and enhanced autophagy [305, 306]. Studies indicate that RSV markedly represses the proliferation and viability of human PCs [306, 307], mediating its effects through the attenuation of various signal transduction pathways including the Hh signaling pathway [308]. In the context of PCSCs, Shankar et al. unveiled the inhibitory effects of RSV that is mediated through the induction of apoptosis by caspase 3/7 activation, attenuation of pluripotency markers (Oct4, Nanog, Sox2, and c-Myc), inhibition of drug resistance gene ABCG2, and downregulation of EMT markers (ZEB1, Slug, and Snail) [309]. Very recently, both in vitro and in vivo evidence have shown the capacity of RSV to reverse GEM-induced stemness, enhance GEM sensitivity, and restrain lipid synthesis in PC cells by targeting sterol regulatory element-binding protein (SREBP) 1 [310]. Being a potent chemotherapy sensitizer, RSV deserves appreciation in the clinical setting.

#### Curcumin

Curcumin (diferuloylmethane), a natural phenolic compound found in Zingiberaceae turmeric, has been extensively studied over a couple of decades for its potential antioxidant, anti-inflammatory, anti-infectious, chemopreventive, and pro-apoptotic properties [311]. In PCs, curcumin has been shown to curb growth, migration, angiogenesis, invasion, and metastasis seemingly through the modulation of various signaling pathways including Akt, NF-κB, and Notch signaling. Specifically, curcumin has been shown to suppress hypoxia-induced proliferation, invasion, and migration as well as EMT progression in PC cells via attenuating Hh signaling pathway [312]. Similarly, curcumin inhibits the proliferation, migration, and invasion of TGF-β1-induced PC cells, induces apoptosis, and tempers with EMT via the inhibition of the Shh-Gli1 signaling pathway [313]. Recently, curcumin was found to resensitize chemoresistant PC cells to GEM through the inhibition of the enhancer of zeste homolog-2 (EZH2)-lncRNA PVT1-c-Myc axis [314] and inhibit GEM-resistant tumor growth both in vitro and in xenograft mouse models. Particularly, curcumin prevented the formation of spheroids via downregulation of several self-renewal-driving genes. EZH2, a histone methyltransferase, is a catalytic subunit of polycomb repressive complex 2 (PRC2) and a central epigenetic regulator of CSC phenotype and function [315]. Through interaction with several lncRNAs, EZH2 modulates EMT and cancer stemness that are commonly associated with drug resistance in PC [314]. Besides the EZH2-lncRNA axis, curcumin was shown to hinder PC cell survival and migration, clonogenicity, formation of the pancreatospheres, and PCSC function by targeting EZH2-miRNA regulatory circuit [316]. Furthermore, curcumin has shown promising therapeutic results in combination with GEM, metformin, and omega-3 fatty acids. In line with this, Ning and group have shown that curcumin and metformin can be effective combinatorial drugs for targeting PCSCs [317]. Data from pre-clinical and clinical models envisage that curcumin is a safe therapeutic agent for the management of PCs owing to its broad spectrum of activities against PC cells, the TME, and PCSCs [157]. Benefitting from these results, various drug analogs of curcumin and/or nanoformulated curcumin have been developed with promising therapeutic outcomes [318, 319].

#### Genistein

Genistein is a natural flavonoid (4,5,7-trihydroxyisoflavone) isolated from soybeans and soy products and has multiple profound anti-cancer effects in various cancers, including breast [320], gastric [321], colon [322], and ovarian cancer [323], primarily through the modulation of Wnt/β-catenin and Hh-Gli1 signaling pathway. The anti-cancer effects of genistein in PC have been attributed to ROS-mediated mitochondrial apoptosis, cell cycle arrest, inhibition of STAT3 proteins, downregulation of MMPs [324], and reversal of EMT [325]. Alteration in miRNA expression profiles, such as downregulation of miR-223 and upregulation of its functional downstream target F-box/WD repeat-containing protein 7 (FBW7) [326] as well as upregulation of miR-34a and concomitant downregulation of Notch-1 signaling pathway [327], causing attenuated cell growth and apoptosis, also accounts for the anti-tumor activity of genistein in PC cells. Overexpression of miR-223 has been shown to govern GEM-induced EMT in PC cells, mediated through the downregulation of FBW7 and subsequent activation of Notch-1 pathway [328]. Expectedly, miR-223 inhibitor and genistein in combination was reported to synergistically inhibit EMT, suppress motility and invasion, and enhance GEM sensitivity of PC cells [329]. Of significance, genistein inhibited cell growth, reduced pancreatosphere formation, and altered the expression of CSC surface markers mainly via the downregulation of the Notch pathway [157]. These findings highlight the potential of genistein in the management of PC, specifically by targeting EMT and PCSCs.

#### Epigallocatechin gallate

Epigallocatechin gallate (EGCG) is an abundantly found polyphenol (flavone-3-ol) in green tea and is widely recognized for its chemopreventive and therapeutic properties in numerous cancers [330]. First reported to affect neural stem cell survival or differentiation [331], studies examining the mode of action of EGCG in PC have determined its robust inhibitory effects on the self-renewal abilities of PCSCs [332]. Specifically, EGCG was shown to inhibit the expression of pluripotency sustaining factors (Nanog, Oct4, and c-Myc) and EMT-TFs and suppress the self-renewal capacity of PCSCs by targeting Hh pathway and TCF/LEF activity. In addition, EGCG suppressed cell proliferation and triggered apoptosis in PC cells by activating caspase-3 and downregulating Bcl-2 and X-linked inhibitor of apoptosis (XIAP) protein. Pharmacologic synergy between EGCG and other phytochemicals and/or traditional chemotherapeutic drugs have been shown to amplify the cytotoxic effects as a whole, targeting tumor bulk cells and CSCs. To this end, combinatorial treatment with quercetin and EGCG was demonstrated to impose synergistic inhibitory effects on the self-renewal capacity of PCSCs by blocking the Shh pathway and TCF/LEF activities [332]. EGCG and phosphodiesterase 3 inhibitor have also been reported to synergize in the inhibition of CSCs properties in PDAC [333]. Moreover, the efficacy of EGCG as a monotherapy or combination with GEM has been reported against PC [330].

Besides these enlisted natural/dietary agents, other compounds such as quercetin and sulforaphane have shown potential therapeutic efficacy against PCSCs. Quercetin, a polyphenolic flavonoid, potently eliminates PCSCs and this effect was more pronounced in the presence of broccoli compound sulforaphane [334]. Similarly, sulforaphane, in combination with different cytotoxic drugs (such as Cisplatin, GEM, Doxorubicin, and 5-FU) had an additive or synergistic effect on PCSCs, suggesting its capacity to increase drug-induced toxicity against CSCs [335]. Moreover, sulforaphane and quercetin were shown to complement the activity of green tea catechins to achieve significant inhibition of PCSC features and PC progression [336]. These encouraging findings imply that a blend of bioactive dietary agents, with complementary activities, possess higher efficacy against PCSCs. Anti-cancer research has also highlighted the benefits of plant-derived functional foods in targeting CSCs. For example, extract of a medicinal plant Geissospermum vellosii (also called Pao Pereira) significantly inhibited PCSC population and tumorigenicity via altering the Wnt/β-catenin pathway in vitro and in vivo [337]. Similar effects were observed following treatment with an extract from the root of the medicinal plant Rauwolfia vomitoria (Rau) [338]. Furthermore, seaweed polyphenols were shown to inhibit radiotherapy-orchestrated EMT and stemness in residual PC cells [339]. Taken together, in view of the multitargeted anti-cancer activities, natural agents, either alone or in combination with conventional chemotherapeutic drugs, present a promising safer approach for the management of CSCs in PC which is of clinical interest. Nevertheless, broad assessment of the pharmacologic landscape is warranted for the clinical translation of these phytochemicals in the management of pancreatic and other cancers.

## Conclusion and future prospects

Significant developments in CSC biology have challenged the traditional classical view of CSCs as a hardwired entity. While considered revolutionary over the past two decades, emerging evidence lends support to the concept that CSCs are not fixed hardwired entities but rather defined transient states governed and driven by temporal and spatial characteristics. However, understanding the CSC plasticity is insufficient given their undeniable role in tumorigenesis, tumor relapse, and metastasis, especially in PC. Therefore, from a therapeutic perspective, curative measures should be designed to target and eliminate the CSC population considering the competency of a single CSC to reconstitute the entire tumor. Emphasis should also be laid on targeting the transient/hybrid cells (non-CSCs) that can reload the CSC pool. Several possible strategies aimed at CSC elimination have been formulated, including (i) a direct selective abolition of CSCs (called targeted therapy), (ii) neutralization of the CSC quiescent phenotype, or (iii) destruction of the CSC niche and/or TME.

Most importantly, therapeutic resistance is heavily contributed to the CSC state; this accounts for the great therapeutic potential of targeting the CSC population in therapy-resistant diseases like PC. With investigations proving the superior efficacy of combinatorial regimen involving the chemotherapy drug and a CSC-inhibitor than monotherapy in an otherwise refractory PC disease, it is evident that CSC-targeting should be an integral part of the overall treatment regime. Early accounts on CSC-driven resistance mechanisms have highlighted several principles that could form the basis of efficient targeting of CSCs. Accordingly, innovative approaches have been proposed for the resensitization of CSCs such as utilizing a combination of drugs targeting ABC transporters, stemness signaling pathways, DDR machinery, immune checkpoints, desmoplasia and fibrosis, and metabolic reprogramming. However, despite the concerted efforts as well as curative and promising results in vitro, efficient clinical translation of CSC-targeting remains unaccomplished. This clinical failure has been attributed, partly, to the multifactorial nature of CSC-mediated therapy-resistance. Besides, in recent years, the hierarchical CSC organization has also been contemplated to drive therapy-resistance and hinder clinical targeting of CSCs. Although research elucidating the relationship between CSC hierarchies and therapeutic resistance is sparse, it is appreciated that the CSC hierarchy represents a suitable target that embodies both susceptible and resistant populations featuring a battery of therapy-resisting mechanisms.

Undoubtedly, clinical translation will be expedited by the development of more sophisticated CSC models that include clinical rigor and by adopting the systems biology approach to identify signaling hubs and molecular effectors of the signaling pathways that are required for CSC survival and maintenance. A more comprehensive understanding of the dosing regimen proportional to the physiological function and an increased efficacy of judiciously designed combinatorial strategies will yield improved therapeutic outcomes and fuel the anti-CSC clinical trials.
